# Benchmarking Successional Progress in a Quantitative Food Web

**DOI:** 10.1371/journal.pone.0090404

**Published:** 2014-02-27

**Authors:** Alice Boit, Ursula Gaedke

**Affiliations:** 1 University of Potsdam, Institute of Biochemistry and Biology, Department of Ecology & Ecosystem Modelling, Potsdam, Germany; 2 Potsdam Institute for Climate Impact Research, Earth System Analysis, Potsdam, Germany; 3 Berlin-Brandenburg Institute of Advanced Biodiversity Research (BBIB), Berlin, Germany; University of Waikato (National Institute of Water and Atmospheric Research), New Zealand

## Abstract

Central to ecology and ecosystem management, succession theory aims to mechanistically explain and predict the assembly and development of ecological communities. Yet processes at lower hierarchical levels, e.g. at the species and functional group level, are rarely mechanistically linked to the under-investigated system-level processes which drive changes in ecosystem properties and functioning and are comparable across ecosystems. As a model system for secondary succession, seasonal plankton succession during the growing season is readily observable and largely driven autogenically. We used a long-term dataset from large, deep Lake Constance comprising biomasses, auto- and heterotrophic production, food quality, functional diversity, and mass-balanced food webs of the energy and nutrient flows between functional guilds of plankton and partly fish. Extracting population- and system-level indices from this dataset, we tested current hypotheses about the directionality of successional progress which are rooted in ecosystem theory, the metabolic theory of ecology, quantitative food web theory, thermodynamics, and information theory. Our results indicate that successional progress in Lake Constance is quantifiable, passing through predictable stages. Mean body mass, functional diversity, predator-prey weight ratios, trophic positions, system residence times of carbon and nutrients, and the complexity of the energy flow patterns increased during succession. In contrast, both the mass-specific metabolic activity and the system export decreased, while the succession rate exhibited a bimodal pattern. The weighted connectance introduced here represents a suitable index for assessing the evenness and interconnectedness of energy flows during succession. Diverging from earlier predictions, ascendency and eco-exergy did not increase during succession. Linking aspects of functional diversity to metabolic theory and food web complexity, we reconcile previously disjoint bodies of ecological theory to form a complete picture of successional progress within a pelagic food web. This comprehensive synthesis may be used as a benchmark for quantifying successional progress in other ecosystems.

## Introduction

Coping with global environmental change demands an improved understanding of ecological succession for ecosystem-based management and restoration [1], [2]. The multitude of species that emerge and vanish during succession form characteristic community patterns which are key to determining ecosystem function and services during successional progress. Hypotheses that explain the successional replacement of species advanced from an early deterministic [3] to a more community-controlled [4] and mechanistic [5] point of view. More recent studies [6]–[8] pointed out that the interplay of successional drivers may result in multiple trajectories, calling for a better reconciliation of successional theory with long-term, empirical measurements. However, direct observation of succession is difficult on land because community assembly often takes decades to centuries.

In contrast, the annually repeated seasonal succession of temperate plankton communities is readily observable [9], spanning 30–100 generations of small organisms dispersed in a nearly homogeneous medium. It is ideally suited as a model system of secondary succession because community assembly during the growing season is largely driven by autogenic processes, passing through characteristic stages in just a few months [10]–[12]. This enables to unravel consequences of abiotic forcing in concert with biotic mechanisms [13] such as predator-prey interactions and competition over comparatively short time scales [14], [15].

Studying this annual cycle of seasonal plankton succession provides new insights for general ecology because key mechanisms of community assembly which lead to species replacements over time are not yet fully understood [10], [11], [16], [17]. Although effects of individual mechanisms such as competition and predation are well understood in lab experiments [18], theoretical food web studies [19]–[21], and some natural ecosystems [22], [23] the overarching principles that govern successional progress are still being discussed [24]–[26]. Previous studies on seasonal plankton succession, e.g. the qualitative PEG (Plankton Ecology Group) model [9], its recent update [27], and other related work in freshwater [21], [28], [29] and marine systems [30] focussed on general patterns at the species or functional group level, e.g. on the plankton composition and the biomass dynamics or life history parameters of selected species. However, the findings from these lower hierarchical levels have rarely been mechanistically linked to system-level processes which can be compared across ecosystems and provide a deeper understanding for successional changes in ecosystem properties and functioning.

Ecological succession at the system level was first qualitatively described by Margalef [31] and Odum [4]. Odum (1969) predicted that functional diversity increases through niche differentiation [32] and the emergence of specialists, in particular because more K-strategists with slower growth and reproduction rates were assumed to partly replace the r-strategists during succession [4]. As a consequence, the system's resource efficiency was predicted to increase by many individuals' improvements in energy and nutrient uptake, assimilation, and allocation, while resource losses by system export were predicted to decrease during succession. This is in line with the resource ratio hypothesis [33], [34] which predicts that more resource-efficient producers dominate resource-limited sites in an increasingly closed system with higher system residence times of energy equivalents and nutrients during succession. Vitousek and Reiner [35] extended Odum's prediction by the “nutrient retention hypothesis” which predicts a balance of nutrient in- and outputs when a steady state in biomass accumulation is reached in late stages of succession. Empirical studies on nitrogen retention in old-field succession [36], streams [37] and temperate forests [38] confirmed this hypothesis, but data from tropical forests did not [39]. Here, we test if these predictions hold for natural temperate plankton systems which never reach an equilibrium state [11], [40].

Based on first principles and individual observations, Odum (1969) further hypothesized that biomass accumulates while mass-specific metabolic activity and respiration decrease as larger consumers with lower mass-specific metabolic rates emerge during succession. He did not verify these qualitative predictions by empirical data, but later studies of freshwater microcosms [41], marine fouling communities [42], and soil microflora [43] provided first quantitative support. However, these studies only dealt with a single metabolic aspect of successional progress (e.g. production or respiration), and did not establish mechanistic relationships between potential drivers, e.g. metabolic parameters and diversity patterns.

A different body of ecosystem theory derived from information theoretics and thermodynamics predicts an increase in information content [44], [45] and organismal complexity [46] during succession. The underlying principle postulates that ecosystems are driven further away from thermodynamic equilibrium by channelling energy into the construction and maintenance of living structures. The system's information content stored in these energy channels is predicted to increase during succession [44], [45]. Thermodynamically viewed, quantitative ecosystem growth is the increase in energy throughput, stored biomass, or network size by the addition of species or functional groups representing the network's nodes. Qualitative growth, in contrast, depends on the internal organization of the energy flows (network links) between such nodes arising from e.g. changes in community composition which affect the energy transfer efficiency and the material residence times at the system level [47].

Odum (1969) verbally linked the thermodynamic concept of system entropy which quantifies the degree of uncertainty in the energy flow patterns in the food web to the information contained in the feeding interactions between its network nodes. He predicted that food chains would become more “web-like”, increasing the system's information content while entropy would decrease by the subsequent elimination of redundant energy flows during succession [4]. In this context, redundancy means that alternative energetic pathways perform similar functions in the food web. Ulanowicz quantitatively tested this hypothesis by developing an index called ascendency [45] which measures the information content of the food web in relation to the magnitude of material and/or energy flows through the system [48]. According to this theory, ecosystems mature during succession by strengthening feeding interactions between functional groups in the food web, thereby minimizing flow redundancy and maximizing ascendency.

However, the only empirically-based study on ascendency along the successional gradient of a single, natural ecosystem [49] known to us did not confirm the predicted trend towards higher ascendency and lower flow redundancy. Hence, the question how quantitative food web complexity should be characterized during succession still remains open and is addressed in this study.

In contrast to information and thermodynamic theory which are based on quantitative flow networks, “classical” food web theory [50], [51] and the related small-world theory define food web complexity from a binary perspective, that is, the presence or absence of feeding links between species or functional groups.

Small-world characteristics are often found in food webs and are characterized by a lower characteristic path length and a higher clustering coefficient than expected by random [52]–[54]. Ecologically, a low characteristic path length means that any pair of species or functional guilds is likely to influence each other through at most one intermediate species in the food web. A high clustering coefficient may indicate triangular constellations of feeding interactions, e.g. when intraguild predation makes two consumers “neighbors” through sharing the same prey. The influences between locally associated nodes are often mediated by well-connected species which represent the network “hubs”. Ecologically, these hubs often have a disproportionally large effect on the community and are therefore termed keystone species [55], [56].

In “classical” food web theory, structural complexity is measured by indices based on the interconnectedness or link density in binary food webs, e.g. connectance (links/species^2^) [57]. The binary view also supports the hypothesis that structural redundancy in food webs is minimized during succession, so binary connectance and hence, structural food web complexity, was predicted to decrease during succession [51]. Opposing this prediction, more recent empirical evidence showed an increase in binary connectance of a soil food web with a growing species number along a successional gradient [58]. The obstacle in reconciling the different concepts of food web complexity is that the connectance based on a binary food web, and the ascendency based on flux quantities are not directly comparable to each other. Hence, a consistent methodology for quantifying food web complexity which unifies food web theory and thermodynamic theory is still missing. Here, we tackle this problem by introducing the flow-weighted connectance and comparing it to the mathematically related ascendency to measure quantitative food web complexity during succession.

The second statement of the thermodynamic theory predicts a successional increase in organismal complexity and defines an index called eco-exergy [46], [59]. Exergy is a concept rooted in physics and engineering that describes the total amount of utilizable energy or “work” in thermodynamic systems. Eco-exergy is its biological counterpart and uses the information content of genetically coded amino acid sequences as a proxy for the work capacity stored in an organism's proteins. Eco-exergy is predicted to increase during succession because selection processes would maximize the work-capacity within living structures as they strive away from thermodynamic equilibrium [60], [61].

Ascendency and eco-exergy offer themselves as universal system-level indices which may be directly compared across ecosystems. However, their comparatively abstract origin in physics and engineering has isolated them from other lines of research in ecosystem theory and calls for an in-depth comparison with other, more ecologically motivated indices.

The described variety of disjoint theories (e.g. ecosystem theory *sensu* Odum, metabolic theory, food web theory, thermodynamics, information theory), ecological perspectives (e.g. taxonomic vs. functional diversity, functional group vs. system level, binary vs. quantitative food web complexity), and the lack of quantitative empirical evidence make the definition and prediction of successional progress difficult. We aim to reconcile these different bodies of theory by confronting them with empirical data from one specific system – Lake Constance (LC). The LC data set provides an exceptional opportunity for investigating successional progress because data on the plankton community are available in weekly to biweekly resolution over 10–20 years. Large and deep LC represents a well-studied model system for secondary succession in pelagic habitats with little allochthonous input [9], [14], [62], [63]. It has recently been demonstrated that the seasonal plankton dynamics in LC provide mechanistic insight into secondary succession as the complex dynamics of its food web were reproducible with high temporal and trophic resolution by a general bioenergetic network model [64]. Hence, we deliberately built upon temporally highly resolved, long-term empirical data from this individual system to avoid artefacts from pooling inevitably coarser cross-system data.

We define successional progress as the mostly biologically driven changes in ecological interactions during the growing season from spring to autumn when vertical mixing intensity is low [65], [66]. We consider spring until the clear water phase (CWP) as early to intermediate stages of succession, and summer until the end of autumn as the late successional stages. For the first time, this study focuses not only on the taxonomically resolved biomass development, but reveals mechanistic relationships between diversity patterns, energetic drivers, resource dynamics and food web complexity during secondary succession.

Secondary succession leads to changes in community functional composition at multiple hierarchical levels (e.g. species-, functional group-, and community-level) which affects the trophic structure and the flows of energy and nutrients through the food web over time. We used four hierarchical levels of food web aggregation by dividing the pelagic community either into 24 functional **guilds** (e.g. small filter-feeding ciliates, see Table 1) as the smallest unit, 8 major functional **groups** (e.g. all ciliates) as an intermediate unit, 5 biotic trophic **compartments** as the coarsest unit (autotrophs, bacteria, herbivores, bacterivores, and carnivores) of trophic organization, or considering the **food web** in its entirety. To avoid confusion, the term “**functional guilds**” refers to the 24-guild (high) resolution and the term “**functional groups**” to the 8-group (intermediate) food web resolution, while the term “**system level**” refers to the entire food web. We investigated the food web across these hierarchical levels and across four trophic levels ranging from bacteria and autotrophic phytoplankton, herbivorous and carnivorous zooplankton to fish.

**Table 1:** The LC food web model comprises 24 functional guilds aggregated to 8 major functional groups.

ID^1^	Name	Description	Size^2^	Diet ID^3^
1	Alg1	Single-cell algae, ++^4^	6	-
2	Alg2	Mostly large, single-cell algae or colonies, +	8	-
3	Alg3	Filamentous blue and green algae, –	5	-
4	Alg4	Diatoms, colonies, filamentous/spiky algae, +	7	-
5	Alg5	Small, coccal algae, ++	3	-
6	APP	Autotrophic picoplankton (cyanobacteria), +	−2	-
7	Bac	Heterotrophic bacteria	−6	PDOM^5^
8	HNF	Heterotrophic nanoflagellates, B^7^	3	6–7
9	Cil1	Small ciliates, B	8	6–7
10	Cil2	Small ciliates, B/H	11	1,5–8
11	Cil3	Medium-size ciliates, H	12	1–2,5,8
12	Cil4	Medium-size ciliates, H	13	1,5,8
13	Cil5	Larger ciliates, O	16	1–2,4–5,8–11
14	Rot1	Small rotifers, B/H	14	1,5–8
15	Rot2	Medium-size rotifers, H/O	15	1–5,8–9
16	Rot3	Large rotifers, O	16	1–5,8–9
17	Asp	Large rotifers, C	16	2–4,8–16
18	Dap	Cladocerans and calanoid copepods, H/O	23	1–16
19	Cyc	Cyclopoids, C/O	20	1–5,8–19^6^
20	Lep	Cladocerans (Leptodora&Bythotrephes), C	26	17–18
21	Fish1	Fish larvae, C	40	14–19
22	Fish2	Juvenile fish, C	42	18–20
23	Fish3	Adult planktivorous fish, C	45	18–20
24	Fish4	Adult piscivorous fish, C	46	18–22

The 24 functional guilds and 8 groups are: Phytoplankton (guild ID: 1–6), Bacteria (ID: 7), Heterotrophic Nanoflagellates (ID: 8), Ciliates (ID: 9–13), Rotifers (ID: 14–17), Herbivorous Crustaceans (ID: 18), Carnivorous Crustaceans (ID: 19–20), Fish (ID: 21–24). ^1^Guild ID. ^2^Size class is log2 (avg. body mass in pgC). ^3^ID of prey guilds. ^4^edibility (++: well-edible, +: less edible, –: edible only for specialists). ^5^Dead particulate and dissolved organic matter. ^6^Links 18→19 and 19→19 describe adult Cyclopoids feeding on juvenile herbivorous Cladocerans (18) and juvenile Cyclopoids (19), respectively. ^7^general diet description (B  =  bacterivorous, H  =  herbivorous, C  =  carnivorous, O  =  omnivorous). For details, please refer to the Methods section.

To quantify successional progress from different ecological perspectives, we used indices derived from the above-mentioned different bodies of theory and accessible from empirical data. We 1.) investigated how and why certain system indices change during succession, and 2.) identified those indices which were most suitable to quantify successional progress in LC and to generalize our findings across ecosystems. More specifically, the indices were used to test the following **three hypotheses (H1-H3)** on successional progress.

**H1** predicts that functional diversity increases while system exports decrease during succession. **H2** predicts that total biomass and average body mass increase, while mass-specific metabolic activity decreases during succession. **H3** predicts that food web complexity, the information content of the food web, and organismal complexity increase during succession. By cross-linking the results from the previously disjoint hypotheses, we establish a coherent picture of successional progress in LC.

The insights gained in this study contribute equally to community ecology and ecosystem theory because of the intertwined control mechanisms driving successional progress at the functional group and the system level. Our findings represent the first quantitative overview of secondary succession under this broad perspective in a specific ecosystem. We discuss advantages and disadvantages of particular indices for quantifying successional progress and by proposing new avenues for generalizing our findings to other ecosystems.

## Materials and Methods

### Ethics statement

No permission was required on this site because Lake Constance (LC) is a large public lake from which water and plankton samples can be taken by everybody. The hydroacoustic monitoring of fish also did not require permission because the animals were not disturbed. Our study did not involve endangered or protected species.

### Study site and measurements

LC is a temperate, large (476 km^2^), deep (mean depth  = 101 m, max. depth 252 m), and warm-monomictic lake north of the European Alps of glacial origin with weak pelagic-benthic coupling, and little allochthonous input into the pelagic zone [67]. Plankton biomass and the factors regulating growth exhibit strong seasonality [9], [68]. The LC data set comprises long-term, high-frequency time series up to 20 years of abiotic conditions (e.g. light, temperature, mixing intensity, nutrient concentrations), species biomasses, production, and the energy and nutrient flows within the food web [14], [65], [69], [70]. The annually repeated, successional cycle in LC is largely driven by autogenic processes during the growing season from March until November [9], [71], [72].

The concentration of **Soluble Reactive Phosphorus** (SRP) from 1995 [73], the **system residence times** (SRT) for C (*SRT_C_*) and P (*SRT_P_*) [74], and the **P**oly **U**nsaturated **F**atty **A**cids (PUFAs) from 2008–2009 within the seston ≤140 μm (Hartwich M., personal communication) were adopted from previous studies.

**Plankton samples** were taken weekly during the growing season and approximately every two weeks in winter at different depths at a central sampling site (max. depth 147 m) in the northwestern arm of the lake. We used the data from the top 20 m layer of the lake which roughly corresponds to the epilimnion and the euphotic zone. The plankton data were evaluated for ten consecutive years (1987–1996) on a standardized time axis [64], dividing the year into 7 phases to reduce the impact of interannual climatic variability: 1. Late Winter, 2. Early Spring, 3. Late Spring, 4. Clear Water Phase (CWP), 5. Summer, 6. Autumn, and 7. Early winter [75]. The interannual variability was much smaller than the seasonal variability [64], [76], [77] which justifies averaging across several years to focus on the overarching successional patterns.

**Plankton abundances** were obtained by microscopic counting [67], [68]. **Body sizes** were estimated by measuring either size frequency distributions of small organisms (e.g. bacteria, heterotrophic nanoflagellates), average cell volumes of intermediate organisms (phytoplankton, ciliates, rotifers), or the individual length of large ones (crustaceans). Species size was converted to **body mass** in units of C using group-specific conversion factors [68]. Fish biomass estimates were inferred from LC catch data of commercially exploited fish species [78] and sonar data [79].

**Production** was measured *in situ* for bacteria, APP [80], and phytoplankton [67], [81]–[83]. Production estimates for zooplankton were gained from a combination of *in situ* and laboratory techniques [69], [84]–[86] and from mass-balanced flow matrices (see below).

### LC food web

Species were assigned to functional guilds sharing the same prey and predator guilds [64], [87] to establish four hierarchical levels of **food web aggregation.** At the highest resolution, the food web was subdivided into 20 plankton and 4 fishes guilds with a total of 109 feeding relationships [87] (Table 1, see Text S1 for details). This **24-guilds resolution** was used to calculate biomass-based indices (e.g. functional diversity) from plankton data averaged over 1987–1996 excluding the fishes as only the adult fishes' total biomass was measured [78], [79]. The intermediate **8-groups resolution** comprises 25 trophic links and 7 detrital flows (Table 2), aggregating 7 major planktonic groups and 1 group of fishes averaged over 1987–1993 [14]: phytoplankton including A*PP* (**Phy**), heterotrophic bacteria, (**Bac**), heterotrophic nanoflagellates (**HNF**), ciliates (**Cil**), rotifers (**Rot**), herbivorous crustaceans (**HerbCru**), carnivorous crustaceans (**CarnCru**), and fishes (**Fish**). Excretion and exudation were gathered in a pool of particulate and dissolved organic matter (**PDOM**). The intermediate resolution was used to calculate the indices involving production or energy flow data (e.g. trophic positions). For improving the visual clarity of the energy and nutrient flow schemes, the 8 groups resolution was further aggregated into **5 trophic compartments**: autotrophs (**Auto,** identical with **Phy**), heterotrophic bacteria (identical with **Bac**), herbivores (**Herb**) comprising Cil, Rot and HerbCru, bacterivores (**Bactv**), and pure carnivores (**Car**). In addition, we defined the dietary group of omnivores (**Omni**) comprising the genera *Eudiaptomus* (part of **HerbCru**) and *Cyclopoides* (part of **CarnCru**) which temporarily develop quantitatively important biomasses and exhibit diet shifts towards carnivory during ontogenesis. The dietary groups were used for depicting the diet-related indices (e.g. predator-prey body mass ratios). Ciliates were categorized into filter feeders and interception feeders based on previous work [88] for determining the functional diversity in respect to their feeding strategies.

**Table 2:** The LC food web model in the 8-groups resolution as the basis of the mass-balanced flow networks.

ID	Name	Diet ID
1	Phyt	-
2	Bac	9
3	HNF	1–2
4	Cil	1–4
5	Rot	1–5
6	HerbCru	1–5
7	CarnCru	1,4–7
8	Fish	5–7
9	PDOM	-1

1 The 7 detrital flows link the dead organic matter of each functional group except of the bacteria back to PDOM which is then taken up by bacteria.

### Energy and nutrient flows

The **trophic structure** was derived from the magnitudes of the energy and nutrient flows between the 8 major functional groups because the flows between the 24 guilds were not directly measurable. These **mass-balanced flows for carbon (C) and phosphorus** **(P)** were established for each of the 7 seasonal phases during 7 consecutive years (1987–1993) from the measurements of biomasses, bacterial, and primary production, group-specific C:P ratios, and from estimates of diet compositions, ingestion, respiration, growth and sedimentation rates [14]. The mass-balance requirement ensures that inputs into each compartment and the entire system equal all respective outputs, considering changes in biomasses as storage flows. The **total system throughput (** ***TST*** **)** is the sum of all compartmental flows *T_i_* over all compartments (*S* = 9, comprising the 8 major functional groups plus the detrital compartment PDOM):
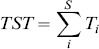
(1)


Note that in all following equations, the value of *S,* being the number of functional guilds or groups, depends on the aggregation level of the food web data on which the indices are based (Table 3). For each compartment, the sum of ingoing flows must equal the sum of all outgoing flows:
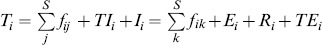
(2)with *S* = 9 functional guilds, 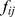  =  trophic and detrital flows from guild *j* into *i* and from *i* into *k* (Table 2), *I*  =  external system input, *E_i_*,  =  system exports, *TI_i_* and *TE_i_  = * biomass storage flows which account for changes in biomass, and *R_i_*  =  respiration [89].

**Table 3:** System-level indices used to test H1–H3.

No.	Index	Abbr.	Food web resolution	Short description (Eq. No.)	Raw data	Hypothesis
1	Functional diversity	*H_bio_*	20 plankton guilds	Shannon's diversity index of relative biomass contributions (3)	Biomass plankton: [67], [68], [76], fish: [78], [79]	H1
2	Succession rate	*σ*	20 plankton guilds	Rate of change in functional guild replacements (4)	see *H_bio_*	H1
3	Food quality	*C:P*	8 functional groups	Ratio of C:P within phytoplankton cells	C:P ratios [73]	H1
4	System residence time C	*SRT_C_*	8 functional groups	Days until a unit of C is lost from the system	Mass-balanced C- & P-flows [14]	H1
5	System residence time P	*SRT_P_*	-	Days until a unit of P is lost from the system	see *SRT_C_*	H1
6	Avg. trophic position	*TP*	-	Fractional trophic level of consumers including fish established from dietary flows (6–7)	see *SRT_C_*	H2
7	Mass-specific metabolic activity	*P_tot_/B_tot_*	7 functional groups	Ratio of total production and total biomass of the plankton community	Biomass plankton: [67], [68], fish: [78], [79]; Production Bac & Phyt: [82], [83], Zooplankton: [69], [84]–[86]	H2
8	Predator-prey body mass ratio	*PPMR*	4 dietary groups	Body mass ratio between consumers and their resources (5)	Body size distributions[68]	H2
9	Trophic transfer efficiency^3^	*TE*	5 trophic compartments	Average production ratio across trophic levels 1–3 in the grazing chain	see *SRT_C_*	H2
10	Weighted connectance	*C_w_*	8 functional groups + PDOM	Interconnectedness and evenness of energy flows in reference to total system throughput	see *SRT_C_*	H3
11	Shannon flow diversity	*H_flow_*	8 functional groups + PDOM	Evenness of energy flows (13)	see *SRT_C_*	H3
12	Weighted char. path length	*D_norm_*	24 guilds + PDOM	Shortest distance between any two nodes weighted by energy flow strength (8–9)	see *SRT_C_*	H3
13	Weighted cluster coefficient	*Q_norm_*	24 guilds + PDOM	Degree of clustering of energy flows around a network hub (keystone consumer) (10–12)	see *SRT_C_*	H3
14	Relative ascendancy	*Asc_rel_*	8 functional groups + PDOM	Network information content normalized by system throughput (16–17)	see *SRT_C_*	H3
15	Specific eco-exergy	*Ex_sp_*	21 (20 plankton guilds + fish)	Organismal complexity in terms of proteome information (18–19)	see *H_bio_*	H3

The **biomass and production pyramids** on ascending trophic levels were derived from the mass-balanced C-flows with primary and bacterial biomass (production) as the basis of the biomass (production) pyramid in the grazing and the detritus chain, respectively. To determine the fractional biomass (production) of a consumer group on a particular trophic level, its biomass (production) was weighted with the relative C-flow (ingestion) between this group and all its resources on the lower trophic levels.

### Indices of successional progress

Below are the indices used to test H1–H3 which we selected because they are accessible from field data and applicable to other types of ecosystems.

#### Indices to test H1: Functional diversity increases and system export decreases during succession

We tested H1 by determining the functional diversity in terms of the evenness of the relative biomass distribution across functional plankton guilds, and the succession rate as the rate of change in functional guild replacements. The system export was approximated by the system residence times of carbon (C, surrogate for energy equivalents) and phosphorus (P, representative of limiting nutrients), and the elemental ratios (C:P) of consumer diets as a proxy of the nutritional quality of food resources during succession.

**Functional diversity** was described by the **Shannon diversity index** [90].
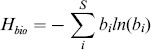
(3)with guild *i*'s relative biomass *b_i_  =  B_i_/B_tot_*, where *B_i_*  =  guild *i*'s absolute biomass, and *B_tot_  = * total biomass of *S* = 20 plankton guilds. *H_bio_* is high for more even distributions of biomass across the plankton guilds and low when a few guilds dominate the community. The speed of community assembly through shifts in the relative importance of plankton guilds was quantified by the **succession rate** [91].
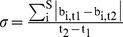
(4)with b_i_  =  relative biomass of S = 20 plankton guilds between two time steps t_1_ and t_2_ (here, t_2_ – t_1_  =  1 day).

The amount of ingested C converted into consumer biomass is constrained by **food quality.** Consumers grow less efficiently on low quality algal food lacking essential compounds such as phosphorus (P) or **P**oly **U**nsaturated **F**atty **A**cids (PUFA) e.g. under nutrient limitation. In LC, P [14] and PUFAs [92] deplete towards summer. The measured cellular **C:P ratios** of phytoplankton [73] often co-vary with concentrations of PUFAs [93] and were used here as an indicator of food quality, e.g. a high C:P ratio in phytoplankton indicates low food quality for herbivores. Bacteria are generally able to maintain lower C:P ratios than phytoplankton due to their higher relative P-content in nucleic acids, higher amounts of membrane-bound P because of higher surface-to-volume ratios, and their competitive superiority in nutrient uptake at low concentration [94], [95].

The **system residence time (** ***SRT*** **)** of elements like C and P measures the mean time between entering and exiting the system and increases in more closed systems. In LC, *SRT_C_* is usually limited to a few days because C mainly exits through respiration and sedimentation of the small plankton organisms with high mass-specific metabolic rates [74]. In contrast, *SRT_P_* may take several days to weeks because P is recycled via the detrital chain and only lost by sedimentation. System residence times of C and P inform about the system export *E* because a higher (lower) *SRT* implies a lower (higher) *E* of the respective element [96].

#### Indices to test H2: Average body mass increases and metabolic activity decreases during succession

We tested H2 by investigating seasonal changes in the measured biomasses and the body mass distributions (i.e. size spectra). To illuminate the mutual influences between the size and the trophic structure of the food web, we linked size-related data to the production-to-biomass ratios (P/B) of functional groups and their trophic positions based on the energy flows between them.

The **biomass** **size spectrum** of the plankton community was determined by allocating all plankton organisms according to their individual body mass into logarithmically spaced size classes. In large pelagic systems, the biomass tends to be approximately equally distributed along the size gradient [97] which also holds for LC [68], [98] and corresponds to a slope of −1 of the normalized biomass size spectrum. This implies that a certain biomass of small organisms sustains approximately the same biomass of larger ones. A more positive (shallower) slope >−1 implies that the biomass of larger organisms exceeds the biomass of smaller ones and vice versa. Hence, the slope informs about the efficiency of the energy transfer from small to large organisms.

The average **predator-prey body mass ratio (** ***PPMR*** **)** was calculated as the weighted geometric mean of the *PPMR*s between the 4 dietary groups in the grazing chain (Auto, Herb, Omni, and Car, see *LC food web* above)
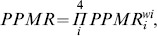
(5)with *PPMR_i_* representing the *PPMR* between carnivores and herbivores, carnivores and omnivores, herbivores and well-edible autotrophs, and herbivores and less-edible autotrophs, respectively. The weights *w_i_* add up to one (*w_1_*  = 0.1, *w_2_*  = 0.1, *w_3_*  = 0.4, *w_4_*  = 0.4) and were established from the mass-balanced flow networks as the fraction of ingestion by the respective dietary group.

Combining the slope of the normalized biomass size spectrum with the *PPMR* enables to estimate the **trophic transfer efficiency (** ***TE*** **)** across successive trophic levels [70], [99]. Alternatively, the *TE* can also be inferred from the production ratio between adjacent trophic levels.

Size is linked to the metabolic activity of organisms by allometric scaling [100], [101]. The **mass-specific** **metabolic activity** was inferred from the production-to-biomass (*P/B*) ratios for each of the 7 major planktonic groups. The system's mass-specific metabolic rate was defined as the total production to total biomass ratio *P_tot_/B_tot_*. In unicellular organisms and small metazoans which dominate the metabolism of the plankton community in LC, activity respiration proportional to production exceeds basal respiration proportional to biomass [102], [103]. Hence, *P_tot_/B_tot_* also informs about the mass-specific respiration (*R*) to biomass ratio as *R* ∼ *P*. Furthermore, metabolic theory predicts that the mass-specific metabolic activity scales with body mass following the allometric scaling law *P/B* ∝ *M^A^* with *M* as body mass and the allometric scaling exponent *A = −0.25* [100], [101], [104]. In LC, it was found that *B* = −0.15 [69].

To calculate the average **trophic position** (*TP*)
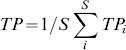
(6)


of *S* = 6 consumer groups in the grazing chain (HNF, Cil, Rot, HerbCru, CarnCru, fish), we used the flow-weighted trophic position [105], [106] of consumer node *i*

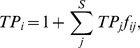
(7)with *S* = 9 (8 major functional groups plus PDOM), *TP_j_*  =  trophic position of resource node *j*, and *f_ij_*  =  relative flow strength (fraction of total C-flow consumed by *i*) between *i* and *j.* Phytoplankton and heterotrophic bacteria were assigned to trophic position 1 and PDOM to 0.

#### Indices to test H3: Food web complexity, information content, and organismal complexity increase

We tested H3 by investigating the relationships between the weighted characteristic path length, the weighted cluster coefficient, the weighted connectance *C_w_*, the ascendency *Asc*, and the eco-exergy *Ex*. All these indices except of *Ex* were derived from the C-flows between the 8 major functional groups and PDOM. *Ex* was calculated from the relative biomass contributions of the 20 plankton guilds and the total biomass of the adult fish. This allows comparing several quantitative measures of internal organization of the food web based either on link attributes (*C_w_* and *Asc*) or on node attributes (*Ex*).

**Food web complexity** was determined by three network indices: the characteristic path length and the cluster coefficient derived from the small world theory [107], and the connectance derived from food web theory [108]. In their original formulation, these indices are calculated from binary feeding interactions. Using the quantitative data on the energy flows in LC, we calculated their weighted counterparts. The motivation to use quantitative network indices is to gain a more realistic picture of the interaction strength between guilds as the quantity of flows varies by several orders of magnitude among nodes and in time.

The small world theory [107] corresponds with food web theory in defining the **characteristic path length**, or “degrees of separation”, and the **clustering coefficient** of a vast variety of natural and man-made networks including food webs [53]. The characteristic path length is the average distance between any two nodes, and the clustering coefficient is the probability that two direct neighbors of a given node are also connected to each other. The term “small-world network” insinuates that any two nodes are likely to be connected through a short path of highly connected network hubs [107]. Small-world networks have intermediate characteristics between regular grids and random networks with high local clustering similar to regular grids, but shorter path lengths more similar to random networks. For food webs, this means that any two species or functional groups are separated from each other by only a few intermediary others over a short path of feeding links [53], [54].

Given that the magnitudes of single flows between the 24 guilds were not directly measurable, we approximated the flow strengths 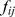 in the 24-guilds resolution by dividing each C-flow in the 8-groups resolution between a consumer and a resource group uniformly between all guilds comprised within the respective resource group. Effects of the level of food web aggregation on the temporal trends of the characteristic path length and the cluster coefficient are described in the *Results and Discussion*.

The **weighted** **characteristic path length** is defined as the average shortest path length
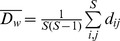
(8)


between all pairs of non-identical guilds *i* and *j* (*i ≠ j*), where *d_ij_*  =  shortest distance or path length between *i* and *j* [109], and *S* = 24 functional guilds. The shortest distance 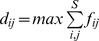 maximizes the sum of the relative flow strength 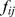 the fraction of the total ingested C-flow between guilds.


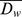
 was normalized as 


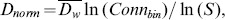
(9)with *Conn_bin_* as the binary connectivity of links *L* per species *S* in reference to the average path length of a random graph with uniform flow distribution which approaches 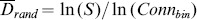 [107].

The weighted average clustering coefficient
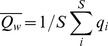
(10), with *S*  =  24 functional guilds and
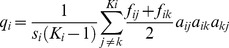
(11)


as the local cluster coefficient of guild *i* is calculated as the average fraction of pairs of “neighboring” guilds *j* and *k* which are directly connected to the focal guild *i* and which are also linked to each other [110] (Fig. S1 in Text S1). In the denominator of *q_i_*, the factor 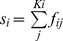 is the total flow strength between focal guild *i* and the total number *K_i_* of all its direct neighbors *j*. The normalization term 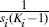 ensures that *0≤ q_i_ ≤1.* Within the sum over *K_i_* direct neighbors *j* and *k, a_ij_* are the binary elements (0 or 1) of the adjacency matrix. This definition weighs closed triplets between neighboring guilds *i, j,* and *k* with the flow strength between *i* and all its direct neighbors. 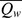 was normalized as
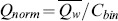
(12)


in reference to the clustering coefficient of a random graph with uniform flow distribution which approaches the binary **connectance** *C_bin_* [52].

In a directed network of *S* nodes and *L* links, *C_bin_  =  L/S^2^* [108] is defined as the ratio between all realized and all possible trophic links. *C_bin_* is a standard measure of how densely species or functional groups are connected by feeding interactions [57]. Natural food webs typically have a binary connectance around 0.1–0.2 [52].

To calculate the **weighted connectance** ***C_w_***, the strength of the C-flows was accounted for in the nominator of *L/S^2^* with *S* = 9 functional groups and *L* = 32 links (Table 2). This novel index is based on Ulanowicz's [111], [112] approach to define the “effective number of connections per node”, hereafter weighted link density, as *Conn_w_*  =  e^0.5*Φ^. In the definition of *Conn_w,_* the system's relative overhead *Φ  =  H_flow_−AMI* is also called conditional entropy [45]. *H_flow_* is **Shannon**'**s flow diversity**

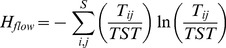
(13)


and *AMI is* the average mutual information
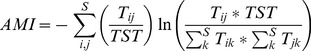
(14)with *T_ij_*  =  absolute magnitude of the energy (C-) flow from group *i* to group *j*. *H_flow_* describes the evenness of the flow distribution relative to *TST*, and *AMI* quantifies the amount of trophic coupling of two groups, also known as the interaction strength [90]. If all links have equal interaction strength, *H_flow_* is maximal, *AMI* minimal, and *Conn_w_*  =  *Conn_bin_*.

*Conn_w_* has been applied to food webs of different size before [113], but it has never been quantified along a successional gradient. In analogy to the definition of *C_bin_  =  L/S^2^  =  Conn_bin_/S*, weighted connectance is defined as
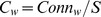
(15)with *S* = 9 functional groups. Note that the links used in the calculation of *C_w_* include all C-flows between the 8 major groups and PDOM, but excluded external in- and outputs, biomass storage flows, and respiration because connectance is by definition [57] an index which only includes feeding links.

The information content and the activity of the system in terms of the distribution and magnitude of the energy flows in the 8-groups resolution was quantified by the **ascendency**

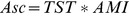
(16)


*Asc* was originally thought to constantly increase with succession [45]. However, a follow-up study [111] suggested that long-term sustainable ecosystems stabilize at intermediate ascendency because systems with very evenly distributed flows ( =  low *Asc*) could drift into chaotic behavior due to the lack of internal constraints, whereas those with very unevenly distributed flows and low redundancy ( =  high *Asc*) may be more vulnerable to external perturbations [111]. An example for the latter is a food web with many highly efficient specialists which is predicted to be less resilient against e.g. species loss. The relative ascendency
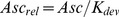
(17)


puts ascendency in relation to its upper bound, the system development capacity K_dev_  =  TST * H_flow_ which is the product of the system's quantitative performance measured by the total system throughput (*TST*) and its internal organization measured by the flow diversity *H_flow_*. The difference *L  =  K_dev_* – *Asc* is the system's overhead [45] and *Φ  =  L/TST  =  H_flow_−AMI* is the relative overhead (also called conditional entropy) which describes the residual uncertainty in the flow patterns of the network. *Asc_rel_* is predicted to increase up to intermediate values of *Asc_rel_  = 1/e  = 0.36* during succession, thereby maximizing the “fitness” *F  =  −k Asc_rel_ ln (Asc_rel_)* of the ecosystem [114]. *F* is considered as a measure of the system's flexibility to undergo structural change defined as the product of the system's order expressed as *Asc_rel_* and the Boltzmann measure of its disorder *−k ln (Asc_rel_),* with *k*  =  Boltzmann's constant. Ecologically, the fitness *F* indicates the adaptive, self-organizational potential of the ecosystem in response to internal changes such as growth and/or the branching of the energy flows as well as to external perturbations during succession [111], [115].

Organismal complexity was determined by a related concept derived from thermodynamics called **(eco-)exergy** ***Ex*** (in g detritus equivalents/m^2^)
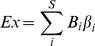
(18)with *S* = 21 guilds (20 plankton guilds and the total biomass of the adult fishes), *B_i_*  =  biomass of guild *i*,in reference to the average *Ex* of 18.7 kJ/g detritus, and *β_i_*  =  group-specific equivalence factors which account for the information storage capacity of the amino acid sequence within proteins which perform work by converting energy in living organisms [116]. *Ex* is defined as the work the ecosystem can perform to maintain order relative to its (hypothetical) unordered state at thermodynamic equilibrium where all elemental components are inorganic and at the highest possible oxidation state [46], [116]. The relevant values of the β*_i_* for the LC food web are: bacteria: β*_i_*  = 8.5, phytoplankton: β*_i_*  = 20; unicellular zooplankton: β*_i_*  = 39; rotifers: β*_i_*  = 163; crustaceans: β*_i_*  = 232; and fish: β*_i_* = 499 [117]. The adult fishes' total biomass was included in calculating the eco-exergy to account for the impact of vertebrates on *Ex* in the plankton-dominated food web of LC. The specific eco-exergy per unit biomass Ex_sp_ is
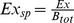
(19)with B_tot_  =  total biomass. Eco-exergy is predicted to increase during succession in concert with the genetic complexity of organisms which may express a greater variety of proteins.

To synthesize our overall results, we selected four key indices *TE, P_tot_/B_tot_*, *H_bio_*, and *C_w_* which are relatively well accessible from empirical data and convey a meaningful picture of successional progress in LC. The phase-wise averages of these indices (cf. Table 4) were normalized by their annual minima and maxima rescaling each value *V* by *Vnorm  =  (V – min V)/(max V – min V)* to yield equal ranges for each index. These indices 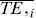, 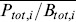, 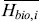, and 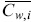, were combined to a composite index 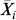 by calculating their arithmetic mean in each phase *i*:
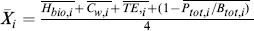
(20)


**Table 4:** Temporal trends in system-level indices of successional progress in LC.

No.	Index	Abbr.	Trend^1^	Early	Interm.	Late	Annual Avg.	Unit	Hypo^2^
1	Functional diversity	*H_bio_*	bi	2.12/2.15	1.66	2.17/2.19	2.04±0.20	bits	H1+
2	Succession rate	*σ*	bi	0.014/0.025	0.023	0.015/0.01	0.014±0.008	d-1	H1+
3	Food quality	*C:P*	down	1034/137	173	357/312	191±106	μgC/µgP	H1+
4	System residence time C	*SRT_C_*	uni	3.2/3.3	10.5	4.8/5.7	5.8±2.5	d	H1+
5	System residence time P	*SRT_P_*	up	3.1/3.8	19.0	10.4/20.8	11.2±7.0	d	H1+
6	Avg. trophic position	*TP*	up	2.40/2.37	2.45	2.53/2.51	2.45±0.08	-	H2+
7	Mass-specific metabolic activity	*P_tot_/B_tot_*	down	0.25/0.24	0.21	0.2/0.15	0.19±0.05	d^−1^	H2+
8	Predator-prey body mass ratio	*PPMR*	uni	3.68*10^2^/4.27*10^2^	1.35*10^4^	2.22*10^3^/4.85*10^3^	4.07*10^3^±4.82*10^3^	-	H2+
9	Trophic transfer efficiency	*TE*	uni	0.20/0.22	0.36	0.28/0.28	0.28±0.06	-	H2+
10	Weighted connectance	*C_w_*	up	0.12/0.16	0.18	0.2/0.21	0.18±0.03	-	H3+
11	Shannon flow diversity	*H_flow_*	up	4.15/3.99	4.35	4.17/4.26	4.26±0.15	bits	H3+
12	Weighted char. path length	*D_norm_*	const	0.74/0.74	0.74	0.75/0.74	0.74±0.002	-	H3+-
13	Weighted cluster coefficient	*Q_norm_*	uni	2.12/2.17	2.39	2.17/2.12	2.20±0.09	-	H3+
14	Relative ascendancy	*Asc_rel_*	down	0.42/0.43	0.37	0.4/0.36	0.39±0.03	-	H3-
15	Specific eco-exergy	*Ex_sp_*	uni	123/103	164	120/145	141±27	unit *Ex*/unit biomass^3^	H3-

Summary of system-level indices describing successional progress in LC. Indices for early (phases 2/3: early/late spring), intermediate (phase 4: CWP), and late (phases 5/6: summer/autumn) succession are averages across the respective phase. Annual avg. is the arithmetic average across phases 1–7. The standard deviation indicates the seasonal variability. Indices 1–5 apply to hypothesis H1, 6–9 to H2, 10–15 to H3. ^1^uni  =  unimodal curve, bi  =  bimodal curve, up  =  upward trend, down  =  downward trend, const  =  constant trend. ^2^Hypo  =  Hypothesis. A “+” (“−“) indicates that the trend of the index supported (opposed) the respective hypothesis. ^3^g detritus equivalents/gC.

In the numerator, we added the reciprocal of the metabolic activity (1-*P_tot_/B_tot_*) to the other indices to be consistent with the directionality of successional progress as predicted by hypotheses H1-H3.

## Results and Discussion

We analyze and discuss the seasonal succession in the LC food web from the functional group to the system level. To integrate structural and functional aspects of successional progress, we used indices derived from ecosystem theory, food web theory, information theoretics and thermodynamics which are generalizable across ecosystems. First, we give an overview of the seasonal biomass, production, and energy flow patterns. Then we present the evidence for the hypotheses H1-H3 to a.) answer why certain system indices change during succession in the observed direction, and b.) identify the most suitable indices for quantifying successional progress in LC which may also be obtained for other ecosystems.

### Overview of biomass and production patterns

**Figure 1:**
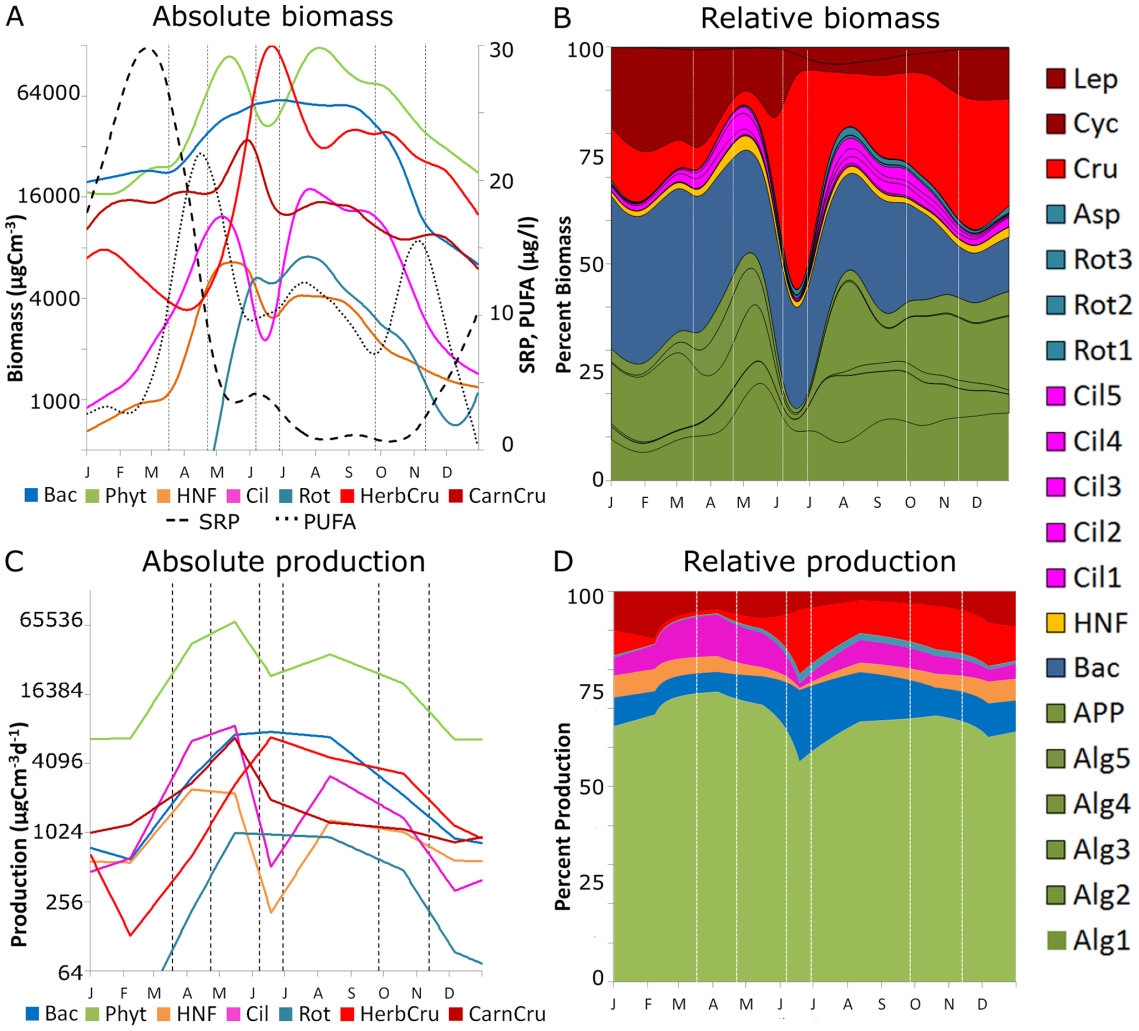
Biomass (A–B) and production development (C–D) during succession. (A) Absolute biomass of the 7 major plankton groups in reference to concentrations of Soluble Reactive Phosphorus (SRP, dashed line, data from 1995, see Methods) and cellular levels of polyunsaturated fatty acids (PUFA, dotted line, avg. 2008–2009, see Methods) within the plankton of size fraction <140 μm in µg/l. (B) Relative biomass of all 20 planktonic guilds (cf. Table 1). (C) Temporal course of the absolute and (D) relative production.

The **absolute** (Fig. 1A) **and relative** (Fig. 1B) **biomasses** of the plankton groups exhibited strong seasonality (Fig. 1A–B, Fig. S2 in Text S1, see animation in Video S1 ) resulting from growth and consumption in the grazing and the detritus chain. Conceptually, the grazing chain is formed by autotrophs (phytoplankton) which are consumed by herbivores (HNF, Cil, Rot, HerbCru) which, in turn, are preyed upon by carnivores (CarnCru, Fish). The detritus chain consists of osmotrophic bacteria remineralizing detritus and preyed upon by bacterivores (e.g. HNF, Cil, Rot). Consumers with mixed diets (Table 1) were allocated partly to the bacterivorous and partly to the herbivorous compartment.

Bacterial biomass steadily increased until autumn. Phytoplankton and the small, unicellular grazers (HNF and Cil) were characterized by a bimodal pattern during succession with almost synchronized spring and summer peaks. The first phytoplankton peak occurred during early succession when density-independent growth was possible due to improved abiotic conditions and high nutrient availability. The small size and lack of defense structures enabled high growth rates for well-edible phytoplankton resulting in the maximal autotrophic to heterotrophic (A/H) biomass ratio (44:56%) during succession in LC. The second peak occurred during late succession when more grazing resistant phytoplankton guilds developed higher biomasses. The biomasses of the metazoan consumers, namely rotifers, herbivorous and carnivorous crustaceans, peaked asynchronously during intermediate to late succession.

The peak of the herbivorous crustaceans and the low biomass of the small phyto- and zooplankton during the clear water phase (CWP) indicate a temporary phase of dominance by daphnids, a generalist keystone consumer. Daphnids represent r-strategists with high reproduction rates enabling a 10–20-fold biomass increase from spring towards the CWP. Their feeding activity on abundant well-edible phytoplankton and on smaller grazers by intraguild predation suppressed their prey guilds' biomasses, resulting in a temporarily low autotrophic-to-heterotrophic (A/H) biomass ratio (A/H  = 18:82%). Towards summer, food shortage and intense grazing pressure from carnivorous invertebrates and fish terminated the daphnids' dominance so that phytoplankton recovered fast and developed summer and autumn blooms. A diverse summer and autumn plankton community established itself until the growing season was terminated by aggravating abiotic conditions in early winter.

Absolute (Fig. 1C) and relative (Fig. 1D) **production** were seasonally correlated with biomass for most guilds. In comparison with its biomass, phytoplankton production gained importance by providing the energetic basis of the largely autochthonous food web and comprising 56–74% of total production in all phases (cf. Fig. S3 in Text S1). Maximal primary production (*PP*) was already reached during the spring bloom (Fig. 1C) when small and fast growing phytoplankton benefitted from high nutrient and light availability and less intensive grazing pressure. Increasing *PP* of mostly high quality phytoplankton during spring enhanced heterotrophic production and led to the mass development of predominately herbivorous crustaceans often dominated by daphnids (Fig. 1D). The resulting comparatively low A/H production ratio (CWP: A/H  = 56:44%) was energetically unsustainable for more than a few weeks so that both absolute and relative phytoplankton production recovered in summer. The absolute amount of *PP* gradually decreased from late summer onwards due to declining nutrient levels, ongoing grazing pressure, and later, also due to aggravating abiotic conditions in autumn and early winter such as deep mixing.

The average ratio between *PP* and bacterial production was high (9:1) because the energy input into the grazing chain considerably exceeded the one into the detritus chain. Bacterial production gained absolute (Fig. 1C) and relative (Fig. 1D) importance during the CWP due to the suppression of phytoplankton and the higher availability of dead organic matter from the spring bloom and the intense feeding activity of grazers. It levelled off at about 15% of total production during summer and autumn.

### Overview of energy and nutrient fluxes

**Figure 2:**
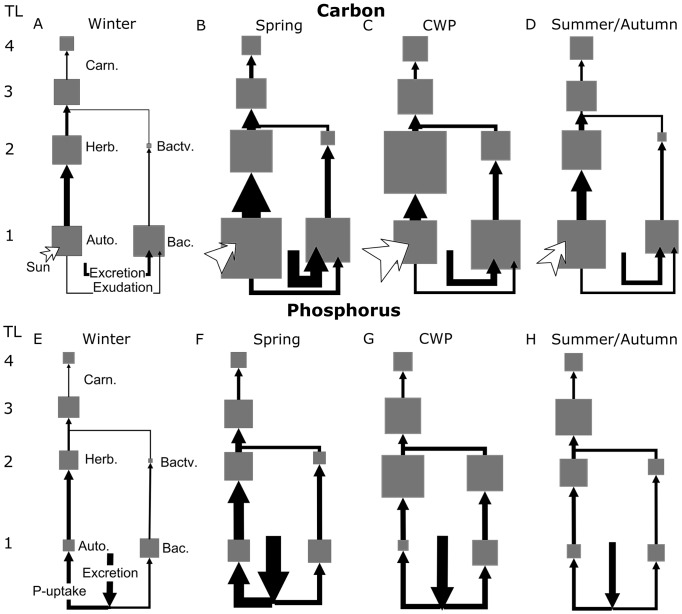
Energy (carbon) and nutrient (phosphorus) flows. (A–D) In-going flows of carbon between major compartments in winter (A), spring (B), CWP (C), and summer to autumn (D). Data from winter (phases 1+7) and summer and autumn (phases 5+6) were pooled to summarize similar flow schemes. Functional guilds aggregated into trophic compartments (see Methods). Auto.  =  Autotrophs, Herb  =  Herbivores, Carn  =  Carnivores, Bac  =  Heterotrophic Bacteria, Bactv  =  Bacterivores. Excretion summarizes the release of dead particulate and dissolved organic matter (PDOM) by phytoplankton exudation, the consumers' feeding and metabolic activities (including sloppy feeding and non-grazing mortality). Arrows widths (compartment areas) are scaled relatively to the square root of the strength of the C-flows in µgC m^-2^ d^−1^(C-content of biomass in µgC m^−2^). (E–H) Phosphorus (P) flows between major compartments in winter, late spring, CWP, and late summer to autumn. Seasonal scaling for P is analogous to C.

The trophic flows in units of carbon (C) (Fig. 2A–D) and phosphorus (P) (Fig. 2E–H) between the five compartments are components of the total system throughput *TST* which is an indicator of system activity (cf. Methods). The largest flow of energy, primary production (*PP*), was low in winter and mostly consumed by overwintering herbivorous crustaceans. During the spring bloom, the C-flows increased more than 10-fold due to the strongly enhanced flows at the bottom of the food web (Fig. 2B). The initially high *PP* of small autotrophs compensated for the considerable losses during trophic transfers and an even greater share of *PP* (up to 90%) than previously thought [26] entered the grazing chain (Fig. 2B–C). An increase in C and P flows similar to the grazing chain was observed in the detrital chain from dead particulate and dissolved organic matter (*PDOM*) towards the bacteria (Fig. 2B and 2F). As bacterivores were less efficient than herbivores in removing biomass from trophic level 1, bacterial biomass increased while phytoplankton biomass decreased during the CWP (Fig. 2C and 2G). In summer and autumn (Fig. 2D and 2H), the C-flow (P-flow) from phytoplankton to herbivores was already 66% (75%) smaller than during the spring peak because resource depletion and predation pressure from carnivores kept the herbivores under control.

Concerning the nutrient flows, P depleted severely on trophic level 1 from the CWP onwards, while it accumulated on trophic level 2–3 (Fig. 2G–H) so that the herbivores had difficulties to gain sufficient P from feeding on autotrophs alone. In response to this, behavioral and species shifts within the herbivorous compartment changed the diet spectrum of predominantly herbivorous consumers towards bacterivory or carnivory (see *Evidence for H2*). In spring, these groups still gained most of their ingested C (91%) and P (83%) by herbivory, whereas carnivory contributed only 7% (P: 11%), and bacterivory a mere 2% (P: 6%) to their diet. These proportions changed markedly after the CWP. In summer, herbivory had already decreased to 79% for the ingested C and even to 45% for P, while carnivory had increased to 15% (P: 40%) and bacterivory to 6% (P: 15%). This means that the proportion of P gained from carnivory increased nearly 4-fold and the one gained from bacterivory more than doubled during succession. Almost all P entering the herbivorous and bacterivorous compartments was transferred to trophic level 3 by predation, so that P was mainly released by the excretion of carnivores (Fig. 2G–H). In addition, mixotrophic phytoplankton (not shown as an extra compartment in Fig. 2) also gained additional P from bacteria in late succession.

In short, the relative flow strengths of C and P were closely correlated during early succession, but increasingly diverged towards late succession due to declining nutrient levels. In response to this, herbivores adjusted their diet by becoming more carnivorous and bacterivorous during late succession, while the detritus chain gained importance as an alternative nutrient source for predominantly herbivorous consumers.

**Figure 3:**
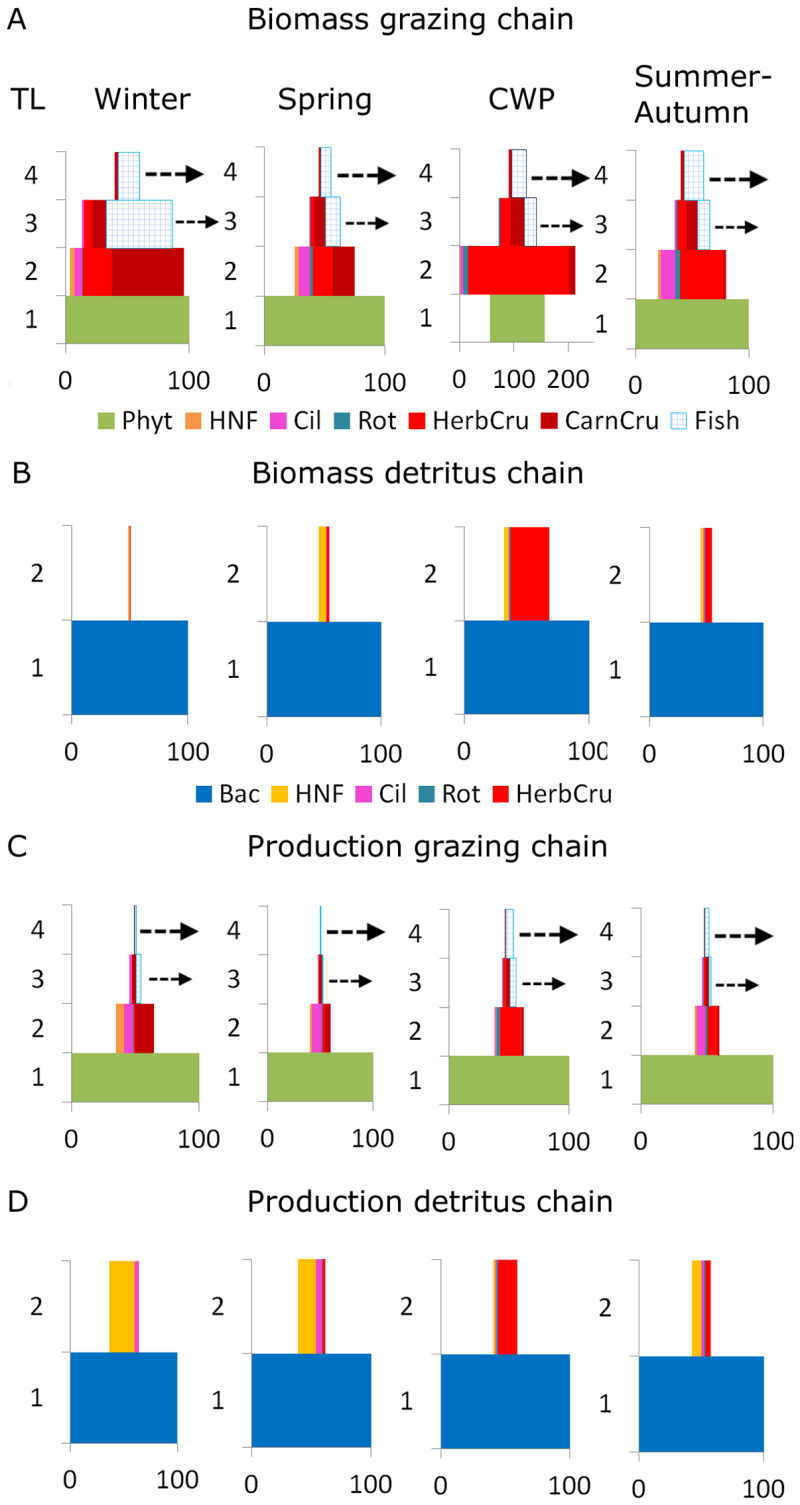
Seasonal changes in trophic structure. (A) The biomass pyramids of the grazing chain and (B) the detritus chain on ascending trophic levels for the 8 major functional groups in units of carbon. Summer and autumn data were pooled to summarize similar distributions. (C) The production pyramids of the grazing chain and d) the detritus chain. Autotrophic biomass and primary production (*PP*) in (A, C) and bacterial biomass and production (*BP*) in (B, D) was set to 100% in each phase. Without this standardization, the ratio between *PP* and *BP* is approximately 9:1 (cf. Fig. 1D). Seasons and groups in (B–D) same as in (A–B). The detritus chain only shows two trophic levels because consumers partly feeding on bacterivores were assigned to the grazing chain. Arrows indicate that fish biomass and production were underestimated because fish biomass is reduced by commercial fisheries in LC (cf. Methods).

Derived from the C-flows (Fig. 2), the **biomass columns** of functional groups on ascending trophic levels (Fig. 3A–B, Fig. S4A in Text S1) link the shifts in functional guild composition to changes in trophic organization during succession. In winter, phytoplankton, herbivorous zooplankton, and fish comprised approximately 1/3 of the total biomass in the grazing chain (Fig. 3A), respectively. In spring, the biomass pyramid was strongest at its base due to the phytoplankton bloom. During the CWP, the high consumer biomass reversed the pyramidal shape of the biomass column. This was also reflected in the detritus chain (Fig. 3B) because the bacterivorous compartment also comprised biomass contributions from predominantly herbivorous guilds which partly fed on bacteria. Bacterial biomass contributed about 10–30% to total plankton biomass in all seasons, whereas the biomass of bacterivores was always small (<5%). This is explicable by the relatively low *P/B* ratio of bacteria in LC [77], [82], [118], especially in comparison to the autotrophs in the grazing chain [81], [83]. In summer and autumn, the previous shape of the biomass pyramid in the grazing chain was restored. The major differences between the spring and the summer/autumn biomass pyramids were caused by shifts in the composition and relative importance of functional guilds within trophic levels (see *Evidence for H1*).

The **production columns** (Fig. 3C–D, Fig. S4B in Text S1) inform about the trophic transfer efficiency between adjacent trophic levels and the energetic structure of the food web. They maintained their pyramidal shape during all phases in the grazing (Fig. 3C) and the detritus chain (Fig. 3D) due to energetic losses by egestion and respiration at each trophic transfer step. The relative production contributions were most evenly distributed during summer and autumn when, despite their lower biomasses, the smaller grazers (Cil, Rot) contributed as much as the herbivorous crustaceans to total production. The detritus chain sustained a more evenly distributed spectrum of bacterivores during summer and autumn.

### Evidence for H1: Functional diversity increases and system export decreases during succession

**Figure 4:**
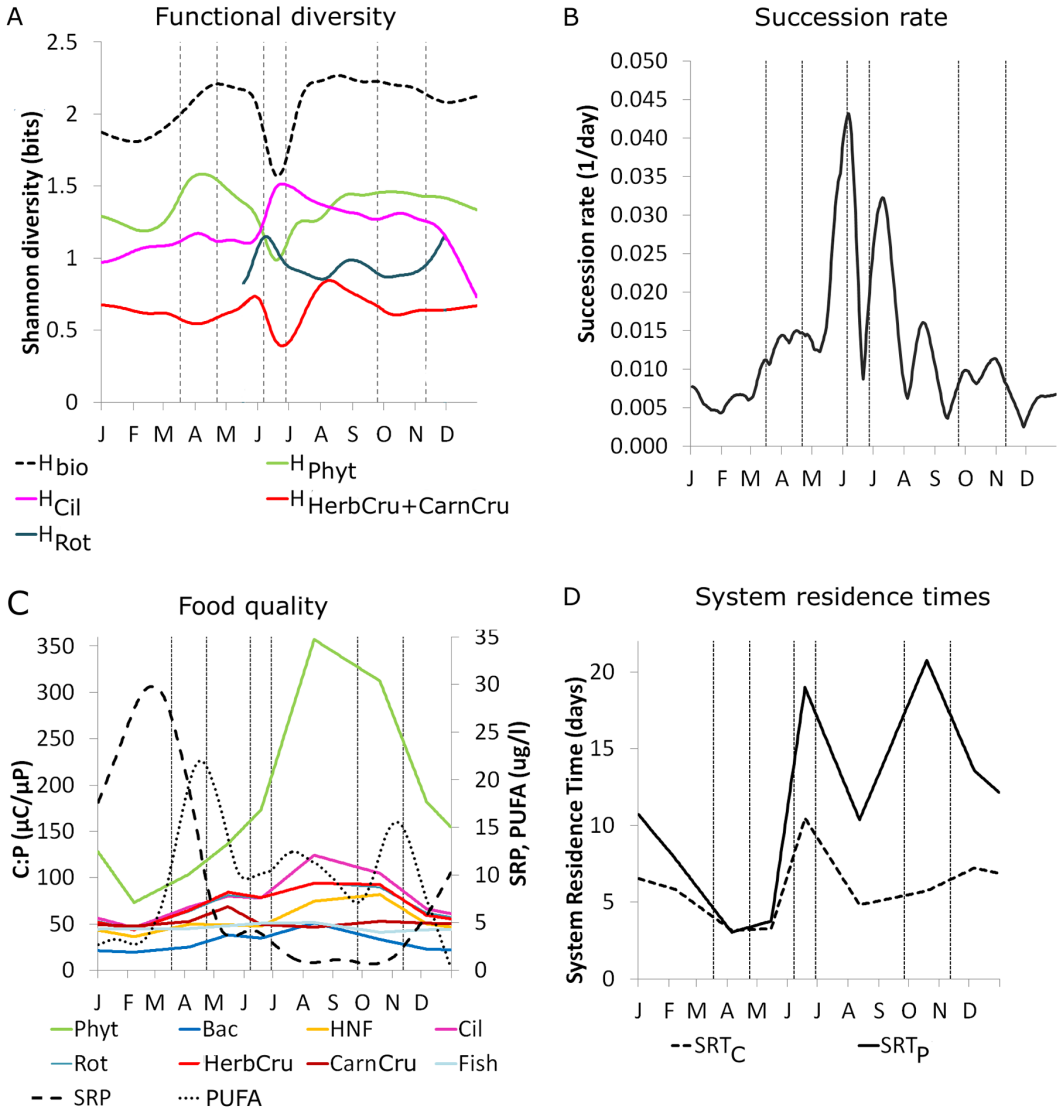
Functional diversity (A), succession rate (B), food quality (C), and system residence times (D). (A) Functional diversity within four major plankton groups: phytoplankton (Phy), ciliates (Cil), rotifers (Rot), and all crustaceans (HerbCru + CarnCru), and system functional diversity *H_bio_* of all 20 plankton guilds (cf. Table 1). Functional diversity of Rot is only shown when Rot biomass exceeded 1% of total biomass. (B) Succession rate *σ* of the 20 functional plankton guilds peaked twice shortly before and after the CWP. (C) C:P ratios of algal and bacterial biomass and food quality of the food ingested by different consumer groups (average across 1987–1993) in relation to phosphorus concentrations (SRP from 1995, dashed line) and cellular levels of polyunsaturated fatty acids (PUFA average 2008–2009, dotted line) within the sestonic size fraction <140 µm [92]. Food quality for herbivores decreased with increasing C:P ratios during succession. (D) System residence times for carbon (*SRT_C_*) and phosphorus (*SRT_P_*). *SRT_C_* and *SRT_P_* were maximal during the CWP due to the dominance of larger crustaceans with slower metabolism and in autumn-winter due to decreasing temperature and on average lower metabolic activity (Fig. 5D).

The system's **functional diversity** *H_bio_* based on the relative biomasses of all 20 plankton groups (Fig. 1B) did not increase monotonously, but followed a more complex, bimodal pattern (Fig. 4A). The bimodality resulted from an initial rise of *H_bio_* during early succession, a sharp decline during intermediate succession (CWP), and a fast recovery followed by a plateau-shaped maximum of 2.19 bits (Table 4) lasting until late autumn during late succession. The increase during early succession was promoted by the initially high resource availability enabling a fast build-up of biomass mainly by fast-growing, well-edible phytoplankton with high functional diversity *H_Phyt_* (Fig. 4A, Fig. S5A in Text S1).

At the functional group level, *H_phyt_* and the crustaceans' *H_Cru_* correlated with the bimodal shape of *H_bio_* because their high relative biomasses (Fig. 1B) contributed the most to *H_bio_*. Sub-dividing the phytoplankton into well-edible and less-edible groups (Fig. S5A in Text S1) revealed that *H_well-edible_* was high in late spring, whereas *H_less-edible_* was high in summer and autumn when species shifts conferred dominance to the less-edible guilds. Among the consumers, both the ciliates' *H_Cil_* and the rotifers' *H_rot_* peaked during the CWP. *H_rot_* showed a lower secondary peak in summer due to a relatively high biomass of a carnivorous genus (*Asplanchna,* Table 1). *H_Cru_* was low during the CWP when only one guild dominated and maximal in late summer due to the emergence of a carnivorous guild (*Leptodora & Bythotrephes)*.

The system's **succession rate** σ (Fig. 4B) based on the daily rate of change in the relative biomasses of the plankton guilds increased 3-fold from winter to early spring when small grazers emerged and another 3-fold at its first maximum in late spring during the transition to the CWP. Intensifying grazing pressure and the subsequent overexploitation of affluent food resources led to major community reorganizations which induced shifts in the functional composition of the phytoplankton (Fig. S5A in Text S1) and grazer community (Fig. 4A, Fig. S5B in Text S1). In particular, the emergence of the predominantly herbivorous, generalist crustaceans suppressed the well-edible, fast-growing phytoplankton which dominated during early succession and promoted the subsequent development of larger and less-edible phytoplankton species in late succession [9], [66]. The temporary dominance of the herbivorous crustaceans was reflected by a sharp drop in σ accompanied by a minimum in H_bio_ (Fig. 4A) during intermediate succession.

The mass-development of generalist herbivores and the subsequently low biomasses of their prey can be interpreted as a pronounced predator-prey cycle which caused a successional setback in terms of functional diversity, but not a reversion of the overall trend towards a new, highly diverse system state. In LC, the pronounced variation in grazing pressure leading to the CWP opens opportunities for less-edible phytoplankton groups to grow and top-level carnivores to feed on the generalist consumers which results in major changes in community composition along a gradient of declining nutrients and food resources.

The extent of the CWP depends on the trophic state of the system and is most clearly expressed in meso- and eutrophic lakes [71], [119] where r-strategist consumers exploit the high food quantity and quality in early to intermediate succession, resulting in the temporary inversion of the biomass pyramid (Fig. 3A) with exceptionally low A/H biomass ratios (≤20:80). Theoretical studies showed that such abrupt transitions in community compositions may occur along successional gradients when fast replacements in functional groups are promoted [120]. A short-term terrestrial phenomenon analogous to the CWP during which a strong predator-prey cycle between producers and herbivores decelerates the succession rate is observed e.g. when migrating ungulates [121] or insect swarms [122] considerably reduce producer biomass. Such changes in grazing pressure are an important driver in terrestrial plant succession [123], especially if the keystone herbivore suppresses the dominant producer group(s) [124], [125].

During the transition from intermediate to late succession, the second peak in σ immediately after the CWP (Fig. 4B, Table 4) indicated another major reorganization of community composition which resulted in a new, functionally more diverse system state where no single species or guild dominated the community anymore. In late summer and autumn, σ returned to spring values, indicating more gradual shifts in community composition until it finally declined towards early winter.

**Food quality** as a potentially growth-determining factor for herbivores is influenced by the seasonally changing stoichiometric balance of carbon and nutrients (e.g. C:P ratio, [73]) and the availability of other essential dietary compounds such as polyunsaturated fatty acids (PUFA) within the tissue of their prey organisms (Fig. 4C). Declining levels of soluble reactive phosphorus (SRP) during succession were accompanied by increasing C:P ratios of maximally 357 (µgC/µgP) in phytoplankton (Table 4) and bacterial biomass, and lower sestonic PUFA concentrations (Fig. 4C). This implies a decline of food quality for the predominantly herbivorous consumers, but not for strict carnivores which remained C-limited throughout succession because the C:P ratios of their diet remained almost constant (Fig. 4C). As a consequence, the production of the herbivores was partly more limited by food quality (e.g. P) than by food quantity (e.g. C) in summer and autumn because their demands for essential dietary compounds were not fully met anymore by consuming low-quality phytoplankton.

In the detritus chain, bacteria maintained substantially lower C:P ratios than phytoplankton due to their different physiological properties in comparison to the eukaryotic plankton [94], [95]. Therefore, predominantly bacterivorous consumers (e.g. HNF) consumed P in excess which was then released by excretion. The bacteria also became a quantitatively important alternative P-source for the predominantly herbivorous consumers. Hence, species gained advantages which covered a larger part of their P (but not C) demand by consuming P-rich bacteria in addition to the P-poor algal food.

The increasing physiological constraints of primary producers and herbivores under nutrient depletion favoured more resource-efficient K-strategists over the r-strategists and, in combination with the enhanced prey resistance to predation in phytoplankton (Fig. S5A in Text S1), led to higher specialization of feeding strategies among consumers (Fig. S5B in Text S1). An increase of selective feeders such as copepods shifted the average diet of the crustaceans towards carnivory (see *Evidence for H2*), thereby exploiting more P-rich resources in autumn. At the top of the food web, an additional guild of comparatively large carnivorous crustaceans (*Leptodora & Bythotrephes*) emerged in response to the high abundances of herbivorous crustaceans (Fig. 1B). The P-rich carnivorous crustaceans, in turn, provided high-quality food for adult fish. These developments at the functional group level led to maximal *H_bio_* at the system level during late succession (Fig. 4A).

The high plateau of *H_bio_* during late succession is in line with a positive impact of enhanced prey diversity on predator diversity also found in other systems [126], [127]. Notably, this development of *H_bio_* was largely independent of system “productivity” in terms of nutrient availability, (primary) production, or total biomass – all of which are often controversially discussed as suitable predictors of community diversity in cross-system studies [128]–[130]. In LC, *H_bio_* remained high until early winter when absolute biomass (Fig. 1A) and production (Fig. 1C) had already declined markedly due to falling temperatures, reduced irradiance and increasing mixing depth. This suggests that neither biomass, nor production is a good predictor of *H_bio,_* and vice versa. Rather, nutrient depletion accompanied by declining food resources and quality enforce changes in functional group composition that lead to higher functional diversity during succession.

More generally, the bimodal shape of *H_bio_* revealed two different, highly diverse system states. The high *H_bio_* during early succession was caused by a relatively even distribution of r-strategist producers, while its maximum during late succession was caused by a diverse and relatively even mixture of both r- and K-strategist producers and consumers across all trophic levels under nutrient depletion. This bimodal pattern is not in line with older predictions of a monotonous increase [4] or a hump-shaped pattern of species diversity [131] during secondary succession. While some empirical studies found a monotonous increase [132], [133], others reported a humped-shaped pattern with a system-specific early [134], intermediate [135] or late maximum [31], depending on the system-specific productivity gradient during succession [136], [137].

Analyzing functional instead of species diversity as we did for LC might help to resolve such conflicting predictions and observations on diversity patterns during secondary succession. However, long-term empirical studies on changes of functional diversity during secondary succession comparable to the LC data set are exceptionally rare. Data from forests support that succession rates are highest during early to intermediate succession [138] and that functional diversity increases with stand basal area as a proxy for successional age [139]. However, such chronosequences have to be interpreted with care because they do not represent continuous time series of the entire food web and are therefore less suitable to elucidate the mechanisms which lead to community change [140]. Multimodal patterns in functional diversity during succession as in LC have been observed in soil microbial and invertebrate communities [141]. However, these belowground patterns depended strongly on detrital dynamics and soil heterogeneities within the local environment. Therefore, they are not directly comparable to the predominantly grazing chain-controlled peaks in *H_bio_* within the pelagic community in LC. Here, we show that these peaks emerge because the effects of ongoing changes in the producers' community composition propagate across several trophic levels and drive consumer diversification during late succession.

The described changes in functional group composition in combination with less sedimentation of nutrient-rich material led to more closed nutrient cycles at the system-level in late succession. Already during the CWP, large amounts of C and P were bound in the crustaceans' tissues. This was reflected in a doubling of the **system residence time** (Fig. 4D, Table 4) of up to 10 days for carbon (*SRT_C_*) and 19 days for phosphorus (*SRT_P_*) due to the relative longevity and slower metabolism of the larger herbivorous crustaceans. From intermediate succession onwards, producers with enhanced nutrient uptake abilities and consumers able to cope with low food supply were selected. This was one reason for the increase in *SRT_P_* during late succession because it is mainly a function of non-grazing mortality [142], but not of metabolic activity as *SRT_C_*. The accumulation of P in larger organisms led to a second peak of the *SRT_P_* of 21 days in autumn, whereas *SRT_C_* levelled off at 6 days, mainly due to the decrease in average body mass after the peak of the herbivorous crustaceans. The different development of *SRT_P_* and *SRT_C_* during succession implies that the system became much more closed for nutrients such as P and less pronounced also for carbon which is always subject to respiratory losses to the atmosphere. Our findings are in line with Odum's (1969) prediction of tightening nutrient cycles, the greater importance of K-selection, and lower exchange rates between organisms and their environment during succession.

A balance of limiting nutrient in- and outputs as predicted by the “nutrient retention hypothesis” [35] was not observed in LC because a seasonal release of nutrients is abiotically induced by the higher vertical mixing intensity and elevated non-grazing mortality towards the end of the growing season. This cyclic, short-term event should not be confused with the mid-term decline of nutrient retention [143] or the long-term nutrient leakage during a retrogressive phase [144], [145] observed in some terrestrial ecosystems.

These insights into diversification by complementary resource use under nutrient limitation are in line with previous theoretical work on plankton communities [146] and studies of secondary succession in terrestrial ecosystems [147]. They suggest that nutrient limitation promotes producer diversification and coexistence during succession. However, these and other previous studies [34], [148], [149] included only primary producers although nutrient-related changes in the biochemical composition of autotrophs may propagate up the food chain [14]. Here, we show how shifts in functional groups and their diet spectra in response to nutrient deficiency promote diversification within the entire food web.

### Summary of evidence for H1

The indices used for testing H1, namely the functional diversity *H_bio,_* the succession rate, the food quality (C:P ratio), and the system residence times for carbon (*SRT_C_*) and phosphorus (*SRT_P_*) combine to a consistent picture of evidence supporting H1. Changes in community composition through species shifts under nutrient depletion led to an increase in functional diversity and a decrease of system export, confirming Odum's (1969) qualitative predictions. We provide new evidence that an ecosystem may develop a high *H_bio_* across several trophic levels in a high- (early succession) and low- (late succession) productive state.

### Evidence for H2: Average body mass increases and metabolic activity decreases during succession

**Figure 5:**
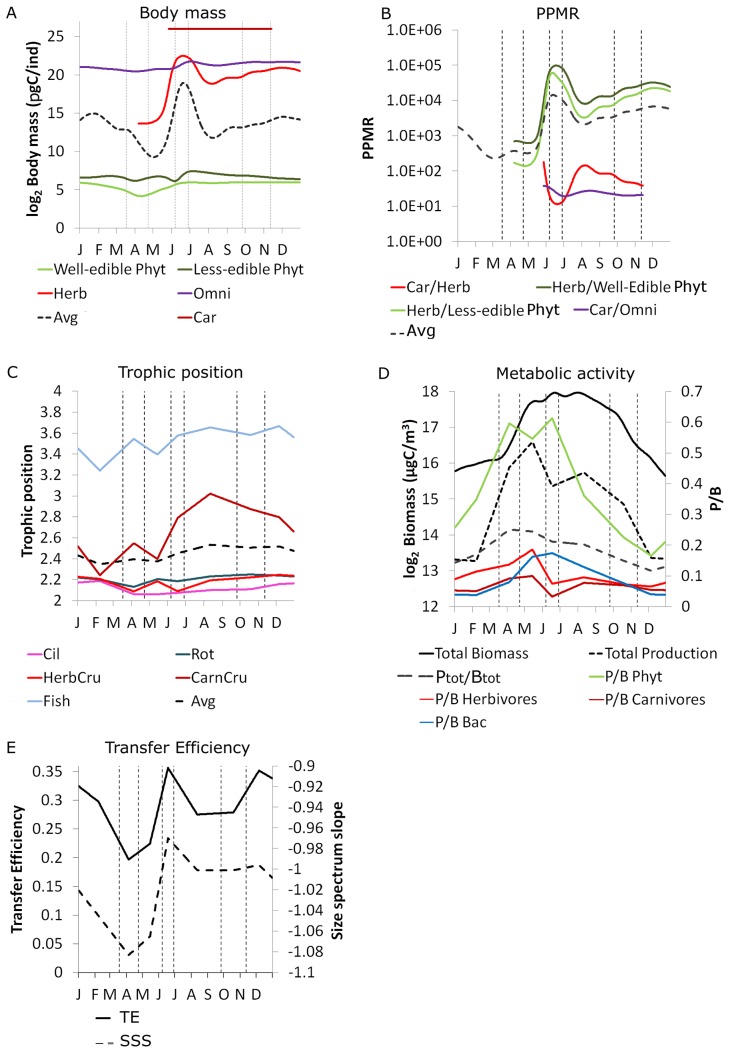
Body mass and metabolic indices. (A–C) Body mass, predator-prey body mass ratio (*PPMR*), and trophic position increased during succession. (A) The biomass-weighted, average body mass of dietary groups changed seasonally due to species shifts within groups. The body mass of large carnivorous crustaceans (*Leptodora & Bythothrephes*) remained constant. Lines in (A) and (B) were drawn only if the group's biomass was >5% of its annual maximum. (B) The increase in herbivore-phytoplankton *PPMR* led to an increase in average *PPMR* by an order of magnitude from spring to summer. *PPMR* was maximal during the CWP due to herbivorous crustaceans' dominance. (C) The average consumers' trophic position established from fractional dietary flows for each group increased with body mass and more carnivorous diets in summer. Bac remained at trophic position 1 and HNF at trophic position 2. (D) Mass-specific metabolic activity (*P_tot_/B_tot_*) of major functional groups and at system level in comparison with total biomass (in µgCm^−2^) and total production (in µgCm^−2^d^−1^). Total production and metabolic activity peaked in early stages of succession before total biomass. (E) The trophic transfer efficiency (*TE*) in units of C within the grazing chain (avg. across trophic level 1–3, cf. Methods) correlated positively (Table S2 in Text S1) with the plankton community's size spectrum slope (*SSS*) and was maximal during the CWP.

Phytoplankton **body mass** increased (Fig. 5A) when the larger and less edible guilds partly replaced the fast-growing, small r-strategists which had dominated during early succession. The small, unicellular herbivores already grew at low temperatures in early spring. A systematic increase in consumer size was observed when the first metazoan rotifers gained importance. The largest increase in body mass of the herbivores by about three orders of magnitude (Fig. 5A) occurred due to the strong proliferation of herbivorous crustaceans which served as the food resource for even larger guilds such as carnivorous crustaceans and fish on trophic level 3–4. This elevated the average body mass of the consumers by an order of magnitude in summer.

Due to the large size difference between the herbivores dominating early (ciliates) and intermediate (crustaceans) succession, the average **predator-prey body mass ratio** (*PPMR*) (Fig. 5B) increased by an order of magnitude, while the *PPMR* between carnivores and herbivores decreased. An increase in average *PPMR* during succession implies fewer trophic transfer steps, a shallower mass–abundance slope [150] and hence, a more even biomass spectrum during late succession. In summer and autumn, the size distribution of the herbivores became more balanced again with the smaller grazers regaining importance relative to the crustaceans. In consequence, the *PPMR* stabilized in autumn around 10^4^:1 between trophic level 2 and 1 and 10^1^–10^2^:1 between trophic level 3 and 2. The average, biomass-weighted *PPMR* within the plankton food web ranged between 3.7*10^3^:1 in early spring and 4.9*10^4^:1 in autumn with maximal values around 1.4*10^4^:1 during the CWP (Table 4).

The average increase in the herbivores' size implied larger prey size ranges and temporarily better protection from predation until carnivorous crustaceans and fish gained in biomass during late succession. In contrast, the increase in the phytoplankton's average cell size had physiological limits because smaller morphotypes have e.g. more favorable surface-to-volume ratios for nutrient uptake than larger ones and suffer less from sedimentation.

An increase in average body mass during succession was also observed in terrestrial vegetation [151]–[153], e.g. when grass is subsequently replaced by shrubs and trees. However, these studies excluded the herbivores and hence, the *PPMR*. More recent studies of average *PPMRs* across different ecosystems [154] found that the *PPMR* between aquatic herbivores and their resources is several orders of magnitudes higher than the one between carnivorous predators and their prey. Our data from LC support these findings. The consequence that the *PPMR* decreases with trophic level is also corroborated by data from terrestrial consumers [155]. Here, we include the primary producers and add evidence that the system-wide, average *PPMR* increases due to the diversification of feeding strategies of the increasingly larger consumers during succession.

The average body mass of planktonic consumer groups and their **average trophic position** (*TP*, Fig. 5C) established from fractional dietary flows were significantly positively correlated (*r_s_*  = 0.51, p<0.001). Including the fish feeding on trophic level four, the average trophic position of the consumers (Fig. 5C) increased during succession (Fig. 5A and 5C) from 2.40 in spring to 2.53 in summer (Table 4), meaning that the biomasses of consumers feeding on trophic level 2 and 3 became approximately equal as reflected in the trophic pyramids (cf. Fig. 3A). This was mainly because predominantly herbivorous groups and in particular, the adult copepods' diet became more carnivorous and because purely carnivorous crustaceans (*Leptodora & Bythotrephes*) emerged.

The system's **mass-specific metabolic activity** was estimated by the total production (*P_tot_*) to total biomass (*B_tot_*) ratio (*P_tot_/B_tot_*). *P_tot_/B_tot_* was low in winter, maximal at 0.24 d^−1^ in early succession when fast-growing, well-edible algae dominated, and declined steadily thereafter (Fig. 5D, Table 4). The early maximum in *P_tot_/B_tot_* was mainly due to the increase in *PP* (Fig. 1C). After a time-lag of about 1–2 weeks, the autotrophs were followed by small grazers with high *P/B* ratios. *P_tot_/B_tot_* already started declining during late spring despite a further increase in temperature and light because of the lower *P/B* of the emerging crustaceans. During summer and autumn, the *P_tot_/B_tot_* was further reduced by the decreasing *P/B* of the phytoplankton and the higher biomass of larger invertebrate carnivores with lower mass-specific metabolic rates. The unimodal development of the mass-specific metabolic activity with its maximum skewed towards early succession reflected both the initially high resource availability and the shift in the size structure from a dominance of small to larger organisms.

Total biomass in LC was still accumulating until mid-summer (Fig. 5D) due to the time lag in the development of the consumer community. Compared to phytoplankton-dominated systems, plants in terrestrial systems generally develop more complex structural tissues and are less grazed by herbivores [26], [156], resulting in overall higher autotrophic to heterotrophic (A/H) biomass ratios during terrestrial succession. Hence, the peak of total biomass in pelagic (terrestrial) systems is caused mainly by the consumers' (producers') biomass. Common to both pelagic and terrestrial ecosystems is that the peak of total system production precedes the peak of total biomass during succession [4], [31], [131], [157] because biomass may continue to accumulate as long as total production exceeds total respiration and system losses.

The **trophic transfer efficiency** *(TE)* between adjacent trophic levels in the grazing chain increased from relatively low spring values of about 0.20 to a maximum of 0.36 during the CWP (Fig. 5E, Table 4) with intensifying grazing pressure by the increasingly active and diverse grazer community. In summer and autumn, the *TE* levelled off at a comparatively high value of 0.28 partly due to declining food quality.

The efficiency of the energy transfer from small to large organisms is determined by *TE* and the number of trophic transfer steps along the size gradient (i.e. the *PPMR*) in pelagic systems because the consumers' trophic position generally increases with size [96], [97]. The energy transfer along the size gradient was enhanced during succession by two distinctive mechanisms.

First, *TE* depends on the consumption and assimilation efficiency influenced by the losses through non-grazing mortality, egestion, and respiration. Losses are high during early succession when only a few grazers with comparatively high egestion rates do not yet fully exploit the affluent food supply. The prevalence of small bodied r-strategists during early succession was reflected in the most negative slope of the normalized biomass size spectrum (*SSS*) and the lowest *TE* (Fig. 5E). The situation was reversed during late succession when larger consumers with lower metabolic (respiration) rates efficiently exploited their entire prey spectrum under the pressure of declining food and nutrient resources. When more biomass and production was sustained on the higher trophic levels, the flow-weighted, average trophic position increased.

Second, the transfer of energy along the size gradient also depends on the *PPMR* between consumers and resources. With *PPMR* already increasing during intermediate succession when the small and highly productive producers were very efficiently exploited by the high abundance of much larger herbivores, energy was more efficiently channeled upwards the size gradient as each unit of consumed energy was converted into biomass of a larger size class in a single trophic transfer step [99].

This explains why the timing of the maximal *TE* coincided with the maximal *PPMR* (Fig. 5B) and the *SSS* (Fig. 5E) in LC. As a result of this energetic “shortcut” in the energy flows from small to large organisms, the high secondary production enabled the inversion of the biomass pyramid during intermediate succession (Fig. 3A).

Due to the linkage between size and trophic position in pelagic systems [96], [97], the *TE* can be deduced either from the production ratio between adjacent trophic levels, or from the *SSS* (Fig. 5E), if the *PPMR* is known [70], [99], [158]. This is relevant because size-related data is often more accessible from measurements than production ratios across trophic levels and offers an alternative method of estimating the *TE* in other pelagic systems. In terrestrial systems, however, the *TE* can be very dissimilar between successive trophic levels and is generally low in the grazing chain (*TE* around 10%) [26] because the herbivores' assimilation efficiencies are low (<10%) [159]. Consequently, less energy reaches the higher trophic levels during terrestrial succession sustaining less consumer biomass in the grazing chain than in pelagic systems.

During late succession, biomass became more evenly distributed along the size gradient with on average larger organisms and the additional, large carnivorous consumers on trophic level 3–4. Consequently, the *SSS* became shallower during succession (Fig. 5E) and the system supported more biomass on the higher trophic levels per unit energy flow.

### Summary of evidence for H2

The size-related indices used to test H2, namely average body mass, average predator-prey body mass ratio (*PPMR*), average trophic position (*TP*), the system's mass-specific metabolic activity (*P_tot_/B_tot_*), and the trophic transfer efficiency (*TE*) confirmed H2. Average body mass increased which, in turn, reduced *P_tot_/B_tot_* during succession. *TP* increased with body size indicating that larger organisms on trophic level 3–4 increased in importance during succession. These developments in combination with the selection for K-strategists entailed changes in trophic and size structure which mechanistically explain the observed increase in the efficiency of the energy transfer along the size gradient during succession. This adds new empirical evidence for the applicability of metabolic theory to natural ecosystems. Our findings are in line with Odum's (1969) prediction of a more efficient use of food resources by on average larger organisms with decreasing *P/B* ratios during succession.

### Evidence for H3: Food web complexity, information content, and organismal complexity increase during succession

The measures of binary food web complexity (Table S1 in Text S1) hardly changed throughout succession because all 24 guilds were present in the food web at almost all times during the growing season. However, the relative importance of the guilds and thus, the strength of feeding interactions, changed pronouncedly. This became apparent only when using weighted network indices which account for the seasonally varying strength of the energy flows between functional groups.

**Figure 6:**
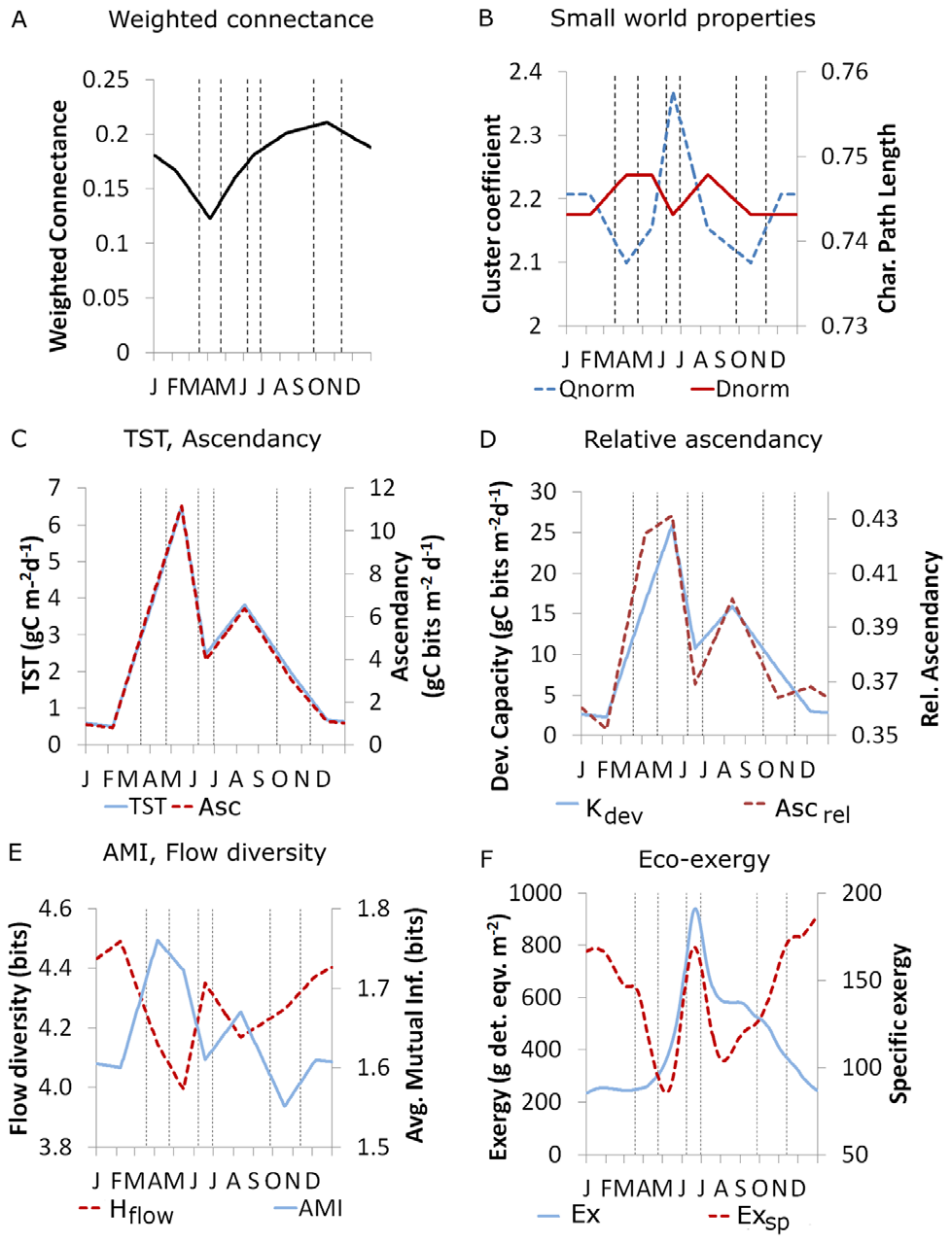
Indices derived from structural food web theory (A–B) and from thermodynamic and information theory (C–F). (A) Weighted connectance *C_w_* increased during succession. *C_w_* was positively correlated with the flow diversity of the trophic flows between the 8 major groups and the detritus pool (Fig. S8 in Text S1). (B) The small-world properties weighted average cluster coefficient *Q_norm_* and characteristic path length *D_norm_*. *Q_norm_*. *Q_norm_* (primary axis) was maximal during times when the keystone consumer (daphnids) dominated the community. Changes in *D_norm_* (secondary axis) were small and opposite to *Q_norm_*. (C) Ascendancy (*Asc*) and Total system throughput (*TST*) peaked during early stages of succession. (D) Relative ascendancy (*Asc_rel_*) peaked in early spring when the average mutual information (*AMI*) (c) was maximal. Development capacity (K_dev_  =  TST * H_flow_) was dominated by *TST* and also maximal in spring. (E) *AMI* decreased during succession while its upper bound, the flow diversity (*H_flow_*) calculated from all flows considered in *TST* (including detrital flows, external in- and outputs, biomass storage flows and respiration, see Methods), increased during succession and exceeded *AMI* during late succession. The difference *H_flow_* − *AMI* is the system's relative (normalized by *TST*) overhead which measures the residual uncertainty in the energy flow pattern (see Methods). (F) Total exergy (*Ex* in g detritus equivalents/m^2^, cf. Methods) and specific exergy (*Ex_sp_* in units of *Ex* per unit of biomass in gC/m^2^) peaked during the CWP due to the high biomass of herbivorous crustaceans.

The **weighted connectance** *C_w_* (Fig. 6A) based on the energy flows between the 8 major groups and PDOM was low (*C_w_*  = 0.12–0.16) in early succession because most C-flows were small and only a few strong ones dominated at the bottom of the food web (Fig. 2, Fig. S6–S7 in Text S1). *C_w_* steadily increased towards its maximum in autumn (*C_w_*  = 0.21, Table 4) because flows which had been weak in spring, e.g. between carnivores and herbivores, gained strength in late succession. *C_w_* was positively correlated with the flow diversity of the same set of energy flows (Fig. S8 in Text S1) because these indices are mathematically related (cf. Methods) and respond similarly to changing flow strengths.

*C_w_* adds a valuable index to quantitative food web theory because its temporal development accurately reflected the increasing interconnectedness of the food web. As opposed to binary food web theory which predicted a decrease of the binary connectance during succession [51], *C_w_* increased steadily (cf. Fig 6A) and expressed the more web-like and more even distribution of the energy flows during late succession. Hence, Odum's qualitative prediction about increasing food web complexity [4] was quantitatively confirmed.

The two **small-world characteristics**, the weighted characteristic path length *D_norm_* and the weighted cluster coefficient *Q_norm_* (Fig. 6B), quantify the shortest distance between any two functional groups and the degree of clustering within the food web, respectively. Both indices were normalized in reference to random networks (see Methods). The temporal average of *D_norm_*  = 0.74 was ¼ units shorter and *Q_norm_*  = 2.20 was more than 2 times higher than expected by random, respectively. This characterizes LC as a small world network, similar to other food webs [53]. The seasonal changes in *D_norm_* were qualitatively opposite to *Q_norm_*, but negligibly small (StdDev  = 0.002) at both the 24-guilds and the 8-groups food web resolution. In contrast, the seasonal patterns of *Q_norm_* differed between the 24-guilds (Fig. 6B) and the 8-groups (Fig. S9 in Text S1) resolution. In the 24-guilds resolution, *Q_norm_* was elevated in winter, minimal in spring and autumn, and peaked during the CWP (max *Q_norm_*  = 2.39, Table 4) due to the many feeding relationships among the neighboring nodes of the keystone consumer (herbivorous crustaceans). As these were bundled together in the 8-groups resolution, the peak vanished in the coarser resolution (Fig. S9 in Text S1). This shows that keystone effects may be masked if the resolution of the energy flow data is too low. In summary, the weighted network indices indicate that the LC food web was more similar to a “small world” during intermediate succession (low *D_norm_*, high *Q_norm_*) and became more complex (high *C_w_*_,_) and less clustered (low *Q_norm_*) during late succession.

The **system-level information content** was measured by the ascendency *Asc* (Fig. 6C) and the relative ascendency *Asc_rel_* (Fig. 6D) in reference to its upper bound, the development capacity *K_dev_*. Contrary to the predictions made by information theory [45], [160], [161], *Asc* and the relative ascendency *Asc_rel_* were maximal during early succession (max. *Asc*_rel_  = 0.43, Table 4) and decreased thereafter (Fig. 6C–D) in concert with their two compounds, the total system throughput *TST* (Fig. 6C) and *AMI* (Fig. 6E). *TST* was maximal in late spring because it was dominated by primary and bacterial production. Most biomass was initially contributed by small organisms with high *P/B* rates which caused strong and yet unbranched energy flows at the bottom of the food web. Due to the strong links between phytoplankton and small grazers *AMI* was maximal in early spring. From the perspective of information theory, this situation represents a strong mutual association between consumer and resource nodes which increases the network information content. However, from the ecological perspective, food web development is still in its infancy passing through a phase of maximal quantitative growth of primary producers with relatively few, but highly active consumer groups.

*AMI* varied less than its upper bound, the flow diversity *H_flow_* (Fig. 6E) which increased during succession. *H_flow_* was high during the CWP and in autumn because the imbalances in the energy flows (few strong links, many weak ones) were reduced when the flows upwards trophic levels strengthened relative to *TST*. The increasing *H_flow_* and the declining *TST* led to an overall decrease in *Asc* (Fig. 6C) and *Asc_rel_* (Fig. 6D) towards late succession.

The usage of *Asc* as a goal function in quantifying successional progress has been critically discussed before [162], [163] because of its sensitivity to food web aggregation which influences *AMI,* and its dependency on *TST*. When defining Asc  =  TST * AMI, Ulanowicz remarked earlier [44] that an initial rise in *Asc* during early succession may be observed when a few species dominate the distribution of the energy flows. The development of the LC food web is an example of this situation because *Asc* peaked early when *TST* was maximal (Fig. 6C), but *C_w_* (Fig. 6A) and *H_flow_* were still low (Fig. 6E). *Asc* is mostly determined by *TST,* if *TST* is large and the variability in relative flow strength among network nodes is low which also holds for early succession in LC. However, not only *AMI,* but also the relative *Asc_rel_* which is standardized by *TST* decreased towards late succession (Fig. 6D). Hence, no matter if the influence of the *TST* on *Asc* was considered or not, its general trend in LC was opposed to the prediction of the original theory [44], [45].

An update of this theory [111], [112] proposed that natural ecosystems exist in a “window of vitality” with intermediate values of *Asc*_rel_ which reflect the “natural tendency for systems to gravitate towards configurations of maximal fitness for change” (Ulanowicz 2002, p. 1890, cf. Methods). That is, ecosystems would develop towards an intermediate state between strongly associated and more flexible interaction patterns in the sense of more or less redundant energy flows. Consistent with this hypothesis, the average *Asc*_rel_ in LC (0.39±0.03) remained close to the predicted maximum fitness of 0.36 [114] because the energy flow patterns were never highly associated (maximal *Asc*_rel_), nor completely evenly distributed (minimal *Asc*_rel_), but always comprised many more weak than strong feeding interactions throughout succession (Figs. S7, S8 in Text S1).

The diversification mechanism leading to the increase in *C_w_* by more evenly distributed energy flows across all four trophic levels was opposite to Odum's expected pruning of redundant energy flows which was supposed to cause a decrease in system entropy [4] during succession. The latter implies a decrease of the system's overhead *H_flow_ – AMI* [89] during late succession. Opposing Odum's (1969) prediction, this difference increased in LC with higher *H_flow_* and lower *AMI* during late succession (Fig. 6E).

The maximum *C_w_* and higher *H_flow_* through functionally similar links [164]–[166] during late succession may imply an insurance effect [167] enhancing response diversity [168] and robustness against disturbances. At least among the phytoplankton, functional redundancy seems likely because its taxonomic diversity, but not its functional diversity (Fig. 4A, Fig. S5A in Text S1), was maximal during late succession in LC (Weithoff et al. *in revision*). Hence, many phytoplankton species in LC probably fulfill a similar function during late succession.

**Organismal complexity** quantified by eco-exergy *Ex* and the specific eco-exergy *Ex_sp_* normalized by total biomass peaked during the CWP (Fig. 6F) because of the high biomass of crustaceans (Fig. 1A) which added more to eco-exergy than the pro- and eukaryotic unicellular plankton organisms due to their 10- fold higher equivalence factor *β_i_* (cf. Methods). The peak of *Ex_sp_* at 164 Ex/g Biomass (Table 4) does not indicate an inflexion point of successional progress because the CWP represents an energetically unsustainable interruption of the successional trend towards higher functional diversity, specialization of feeding strategies, and the increasing importance of K-strategists. In summer, *Ex,* but not *Ex_sp_,* was considerably higher than in spring because of the higher total biomass. *Ex_sp_* was elevated during winter because of the high relative contributions of overwintering crustaceans (26%) and fish (18%) to the overall lower total biomass.

Although some empirical studies [116], [169]–[171] support the increase of eco-exergy during succession, our results do not confirm these findings. Rather, they question the usefulness and the generality of the concept underlying the organismal hierarchy inherent in *Ex_sp_* [172] and its dependency on β*_i_*. The latter do not define organismal complexity based on ecological traits or roles at the phenotypic level, but solely on genome- or proteome related criteria [117], [173]. A peak of eco-exergy during late succession may only occur in systems where organisms with higher *β_i_* replace others. This, however, is not a universal characteristic of successional progress which may also be largely driven by structurally “simpler” organisms as in LC.

### Summary of evidence for H3

The indices used to test H3 did not unambiguously support H3. Food web complexity measured by the weighted connectance (*C_w_*), but neither information content measured by ascendency (*Asc_rel_*) nor organismal complexity measured by eco-exergy (*Ex_sp_*) increased during succession. *Asc_rel_* was maximal when a great share of the energy flow was channeled through a few strong links during early succession, whereas *Ex_sp_* peaked when genetically more complex organisms dominated the community during intermediate succession. Hence, the latter two indices did not support earlier predictions based on information [45] and thermodynamic [46] theory, and did not combine with *C_w_* and the other indices used to test H1–H2 to a consistent picture of successional progress. *C_w_* was a suitable index for assessing the magnitude, evenness and increasing interconnectedness of the energy flows during succession. This supports the usage of flow-weighted network indices for quantifying food web complexity during succession. The increase in *C_w_* is in line with Odum's (1969) prediction of a more “web-like” structure of the energy flows within the food web, but opposes his related prediction of decreasing system entropy due to the observed increase in flow diversity in LC.

### Cross-linking indices

**Figure 7:**
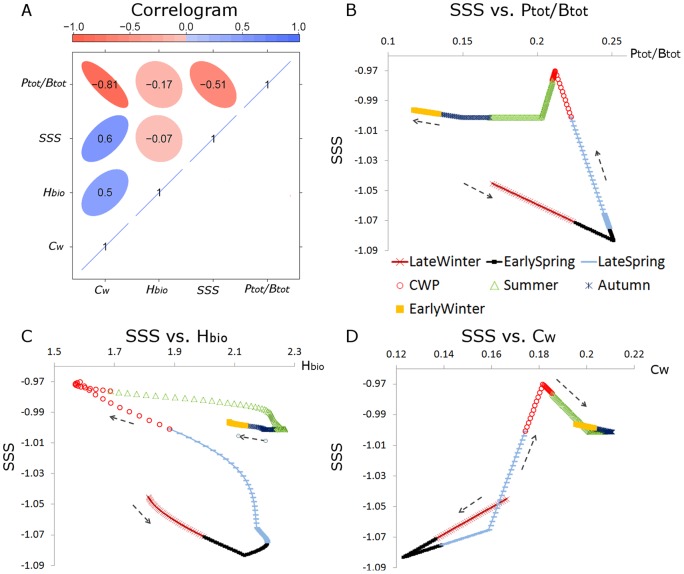
Pair-wise correlations of key indices relevant for H1-H3. (A) Correlogram of four system-level key indices: *P_tot_/B_tot_*  =  system's mass-specific metabolic activity, *H_bio_*  =  functional diversity, *C_w_*  =  weighted connectance, *SSS*  =  Slope of the normalized biomass size spectrum. Blue (red) ellipsoids indicate positive (negative), and narrow (wide) ones indicate strong (weak) Spearman correlation coefficients. Note that the correlation coefficients between *P_tot_/B_tot_* and *H_bio_* (corr.  = −0.17) as well as between *SSS* and *H_bio_* (corr.  = −0.07) were comparatively weak. B–E) Detailed trajectory of pairs of indices with comparatively strong correlations shown in (A). The dashed arrows indicate the direction of the successional trajectory. The distance between the data points increases with the rate of change of the dynamics (approx. 1 data point per week). (B) During early succession, *P_tot_/B_tot_* was maximal and *SSS* minimal due to the fast and density-independent growth of small producers and grazers. Towards intermediate succession, *P_tot_/B_tot_* decreased with increasing body mass, leading to the maximal (shallowest) *SSS*. In late succession, *SSS* decreased again with more evenly distributed biomasses along the size gradient, accompanied by decreasing P/B due to aggravating abiotic conditions. (C) The correlation between *H_bio_* and *C_w_* was mostly positive along sections of the successional trajectory, but temporarily interrupted by a short phase of low functional diversity during intermediate succession (clear water phase). (D) *C_w_* increased while *SSS* became shallower (less negative) with the enhanced energy transfer from small to large organisms during succession. Note that *C_w_* continued to increase in summer and autumn when the slope approached −1 due to the more evenly distributed size classes along the size gradient.

Subsequently, we compare four system-level indices to shed light onto the mechanistic relationships between them. We selected the functional diversity *H_bio,_* the system-wide metabolic activity *(P_tot_/B_tot_),* the slope of the normalized biomass size spectrum (*SSS*), and the weighted connectance *C_w_* because a.) they are easily calculable from empirical data, b.) they cover relevant aspects of successional progress predicted by hypotheses H1-H3 to allow an assessment from different ecological perspectives, and c.) their magnitudes can be directly compared across ecosystems. The Spearman correlation coefficients (*r_S_*) between the time series of the four selected system-level indices indicate a number of strong interdependencies between them (Fig. 7A, Table S2 in Text S1).

Metabolic theory assumes a negative relationship between an organism's body mass and its mass-specific metabolic activity. Consistent with this theory, we found a strongly negative correlation (*r_S_*  = −0.51, p<0.001) between *SSS* and *P_tot_/B_tot_* (Fig. 7A and 7B). The temporal trajectory of *SSS* vs. *P_tot_/B_tot_* (Fig. 7B) shows that *P_tot_/B_tot_* initially rose fast when the average body mass was small (most negative *SSS*) during early succession and decreased steadily with increasing average body mass (less negative *SSS*) from intermediate succession onwards. The initial rise in *P_tot_/B_tot_* was mainly caused by the high growth rates of the phytoplankton entailing a positive correlation between *P_tot_/B_tot_* and primary production (*PP*) (*r_S_*  =  +0.79, p<0.001, Table S2 in Text S1). Although *P_tot_/B_tot_* was already declining during intermediate succession when larger herbivores dominated the community (least negative *SSS*), it was still higher than predicted by the idealized, allometric scaling exponent of −0.25 [100] (Fig. S10 in Text S1) until food quantity and quality declined in late succession. This metabolic “overshoot” indicates that large-bodied organisms were temporarily sustained by the high metabolic activity of their small-bodied resources. This was possible because a.) the phytoplankton reached maximal growth rates being far from their carrying capacity under severe top-down control and b.) the size dependency of metabolic rates in plankton communities is comparatively weak [69], [104], [174].

The negative relationship between *SSS* and *P_tot_/B_tot_* (*r_S_*  = −0.51, p<0.001, Fig. 7A–B) and the positive correlation between *SSS* and *TE* (*r_S_*  =  +0.92, p<0.001, Fig. 5E) led to a negative correlation between *TE* and *P_tot_/B_tot_* (*r_S_*  = −0.57, p<0.001). More generally, the interdependent temporal development of *P_tot_/B_tot_*, *SSS,* and *TE* implies that a certain amount of autotrophic biomass may sustain a similar amount of heterotrophic biomass (Fig. 3A), if the producers' *P/B* considerably exceeds the consumers' *P/B* and *TE* is sufficiently high.

These size-related indices (H2) are linked to the diversity-related indices (H1) and those of food web complexity (H3) through the correlations between *SSS, H_bio_,* and the weighted connectance *C_w_*. The bimodal pattern of *H_bio_* (Fig. 4A) was largely independent of the average body size so that the correlation between *H_bio_* and *SSS* (*r_S_*  = −0.07, p = 0.27) was non-significant. The correlation between *H_bio_* and *P_tot_/B_tot_* (*r_S_*  = −0.17, p<0.01) was weakly negative (Fig. 7A) because *H_bio_* increased while *P_tot_/B_tot_* decreased from early to late succession (Fig. 5D). Notably, *H_bio_* was positively correlated to the bimodal shape of *PP* (*r_S_*  =  +0.39, p<0.001, cf. Fig. 1C, Fig. 4A), but independent of its amplitude because high values of *H_bio_* occurred at high (early succession) and low (late succession) absolute values of *PP*.

In contrast to *H_bio_*, *C_w_* is by definition (cf. Methods) insensitive to biomasses, but responded strongly to the increasing strength and evenness of the energy flows between functional groups. This is why *C_w_* developed differently from *H_bio_* and increased steadily towards late succession. In consequence, the correlation between *H_bio_* and C_w_ was only moderately positive (*r_S_*  =  +0.5, p<0.001, Fig. 7A): The initially positive relationship temporarily changed sign during intermediate succession (CWP) due to the short phase of low *H_bio_* (Fig. 4A) which did not affect *C_w_* (Fig. 6A and Fig. 7C). Hence, only *C_w_* indicated successional progress towards more evenly distributed energy flows between functional groups.

The different temporal developments of *H_bio_* and *C_w_* highlight the suitability of each index for characterizing specific aspects of successional progress. *C_w_* responded more slowly and was more suitable to capture long-term successional trends than *H_bio_* because the development of the more evenly distributed energy flows upwards trophic levels took longer than the fast changes in functional group composition which *H_bio_* accurately reflected as two peaks during early and late succession. This holds true even though *H_bio_* was available in a higher (20 plankton guilds) resolution than *C_w_* (8 major groups), showing that even a comparatively coarse quantitative resolution was sufficient to shed light onto successional food web dynamics. We therefore suggest the usage of *C_w_* to complement *H_bio_* because the energy flow patterns represent an integral part of the system's functional diversity and process rates often proved to be more informative than biomasses for explaining the influence of system-level properties on ecosystem functioning [77].

Given that *C_w_* was a more consistent indicator of successional progress than *H_bio_*, we looked more closely at the strongly positive correlation between *SSS* and *C_w_* (*r_S_*  =  +0.6, p<0.001, Fig 7A, D). During early succession, *C_w_* and *SSS* were minimal (Fig. 7D) because the small producers generated a high amount of *PP* which did not contribute much to *C_w_* because a.) *C_w_* is standardized by the total system throughput (*TST)* and b.) the energy flows were not yet efficiently transferred upwards trophic levels until the consumer community responded to the spring bloom. Towards intermediate succession, *C_w_* rose in concert with *SSS* because most of the *PP* was consumed by the herbivorous guilds through a few, strong energy flows. This implied an enhanced energy transfer from small to large organisms. As opposed to *H_bio_*, *C_w_* and *SSS* increased further during intermediate succession because the well-connected, generalist crustaceans appropriated most of the energy flows and formed a cluster (Fig. 6B) with their various prey guilds. During late succession, *SSS* approached −1 while both Cw and *H_bio_* were maximal. This implied that numerous consumers on higher trophic levels exploited their entire prey spectrum well, so that the high consumption efficiency reduced non-grazing mortality and sustained many large organisms. Consistent with the negative correlation between *SSS* and *P_tot_/B_tot_* (Fig. 7B), we also found a negative correlation between *C_w_* and *P_tot_/B_tot_* (*r_S_*  = −0.81, p<0.001, Fig. 7A) because *C_w_* was high when larger consumers with lower mass-specific metabolic rates drew more energy towards the higher trophic levels.

Overall, the correlations between the key indices *H_bio_*, *P_tot_/B_tot_*, *SSS* and the closely correlated *TE*, and *C_w_* indicate that the highest food web complexity (max. *C_w_*) was realized in the less productive months (low *P_tot_/B_tot_*) during late succession due to the most even distribution of the energy flows (max. *C_w_*) and an efficient energy transfer from small to large organisms and across trophic levels (high *TE*). This resulted in the most even biomass distribution along the size gradient (*SSS* close to −1) within the functionally most diverse food web (max. *H_bio_)*.

**Figure 8:**
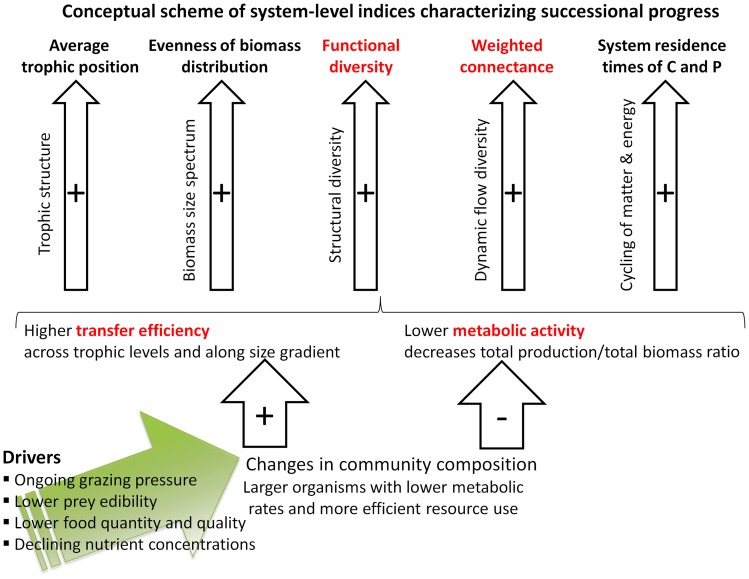
Conceptual scheme linking indices of successional progress. Several drivers of successional progress induce changes in community composition during succession in LC. Higher average body size entails lower system metabolic activity and respiration, while the feeding activities of the more diverse and more specialized consumer community combined with lower non-grazing mortality result in a more efficient exploitation of food resources. These changes in size, diet, and trophic structure enhance the efficiency of the energy transfer towards higher trophic levels and along the size gradient. With more energy reaching larger consumers and the higher trophic levels, biomass becomes more evenly distributed along the size gradient in a functionally more diverse and more complex food web with more closed energy and nutrient cycles. Four key indices (i.e. the transfer efficiency across trophic levels *TE,* the system metabolic activity *P_tot_/B_tot_*, the functional diversity *H_bio_*, and the weighted connectance *C_w_*) are marked in red and combined to a composite index of successional progress (Fig. 9).

**Figure 9:**
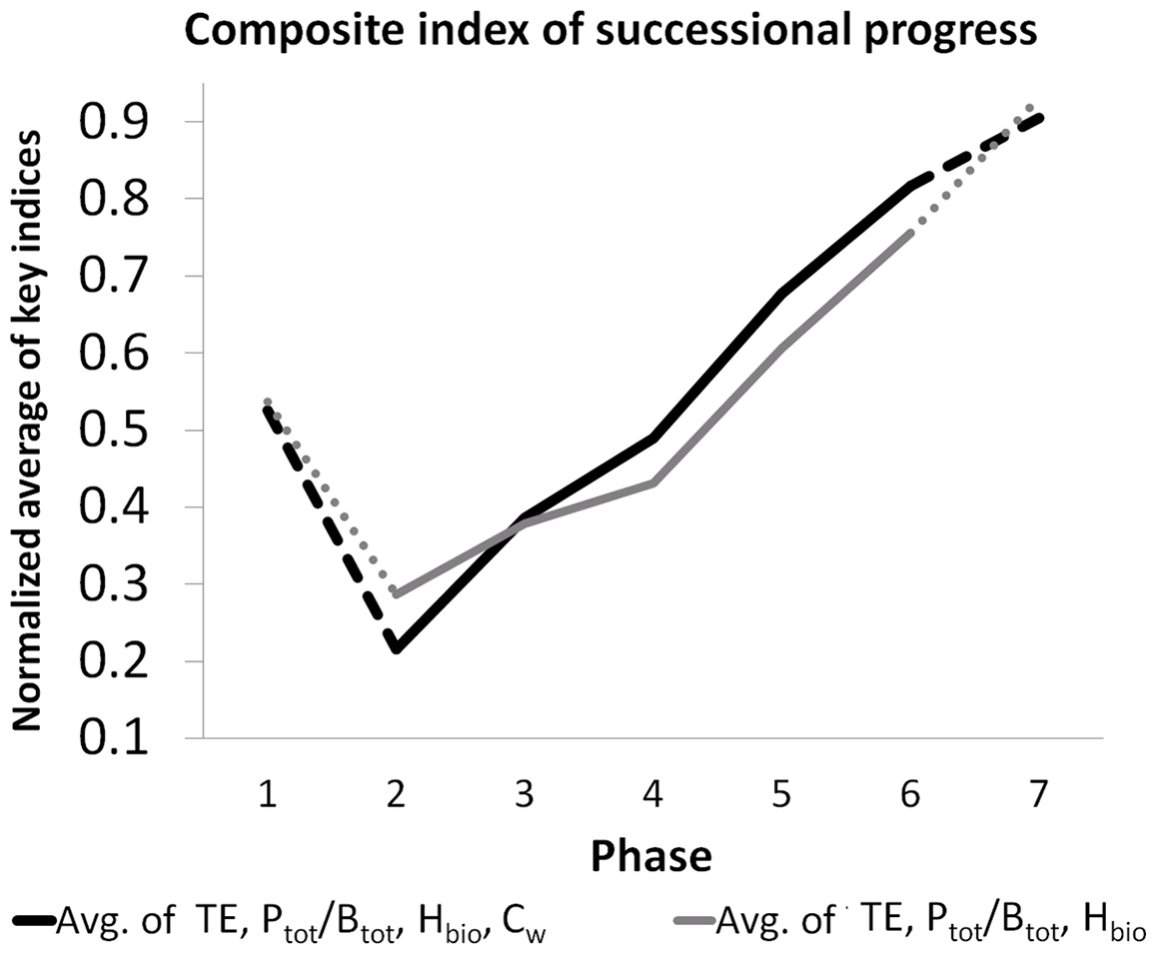
Composite index of successional progress. The composite index based on the average of the four normalized indices *TE, P_tot_/B_tot_*, *H_bio_*, and *C_w_* (black line) increases approximately linearly during the growing season from phase 2–6. The result is similar if *C_w_* is excluded from the calculation (gray line). Higher values during the winter phases (dashed/dotted part of the lines) are caused by the very low *P_tot_/B_tot_* values which are due to the influence of abiotic forcing rather than biotic processes.

Based on our results, the drivers and key indices of successional progress are conceptually integrated (Fig. 8) by the transfer efficiency of energy and the metabolic activity as the central elements which link the size structure to the trophic structure of the increasingly diverse and complex food web. The normalized average of four selected key indices *TE*, *P_tot_/B_tot_*, *H_bio_*, and *C_w_* (cf. Eq. 20) increases monotonously during the growing season (Fig. 9) and represents a quantitative measure of successional progress which is comparable across ecosystems. Including *C_w_* results in a steeper slope from phases 3–4 because the high connectivity (large prey range and resource monopolization) of the herbivorous crustaceans during the CWP enhances *C_w_*, while from phases 6–7 towards winter, *C_w_* decreases and hence, the slope becomes shallower again. The inclusion of *C_w_* in the calculation accounts for the role of the energy flow patterns in the definition of successional progress, but the composite index is still a robust measure even if energy flux data is unavailable.

## Conclusions

Our results indicate that successional progress within the plankton community of Lake Constance (LC) was quantifiable by generalizable system-level indices (Table 4), passing through predictable stages from early to late succession. By drawing on highly resolved long-term empirical data and by establishing cross-references between these indices, we shed light onto the mechanisms driving changes in community composition at the functional group level and how these changes are reflected at the system level. We formulated three hypotheses H1-H3 derived from ecosystem theory which had previously been only qualitatively expressed [4] and never consistently investigated along successional gradients in natural ecosystems [45], [46]. H1-H3 were quantitatively tested and mostly confirmed. By embedding aspects of functional group composition and diversity (H1) into the context of metabolic activity (H2) and food web complexity (H3), we reconcile previously disjoint bodies of ecological theory and provide a set of generalizable indices accessible from empirical data.

The LC data reflect the successional progress in temperate, large open water bodies (both limnetic and marine) of intermediate trophic state. Nonetheless, our insights into the mechanisms underlying these patterns are generalizable to a much wider range of ecosystems. We propose that the temporal developments of *H_bio_, TE*, *P_tot_/B_tot_*, and *C_w_* combine to a suitable set of indices to characterize successional progress across ecosystems. Seen in their entirety, the combination of several interdependent indices turned out to be much more powerful to explain successional progress than relying solely on the temporal development of isolated indices. This is especially relevant if the available time series are not as well resolved as the LC data set because short-term non-equilibrium dynamics such as during the CWP may interrupt temporal patterns of successional progress.

Going beyond Odum's descriptive “strategy of ecosystem development” [4] by the quantification of system-level indices and by gaining new insights into the directionality and characteristics of successional progress from the functional group to the system-level, we derive the following conclusions:

1. Successional progress leads to higher average body size, lower system metabolic activity, higher efficiency of the energy transfer to higher trophic levels and along the size gradient, higher functional diversity, more closed nutrient cycles, a more even biomass distribution across size, higher specialization of feeding types at several trophic levels, and higher food web complexity in terms of increasing redundancy and evenness in the energy flows between functional groups.
2. Multiple peaks of functional diversity are possible and explicable by different system states arising from diversification mechanisms under nutrient limitation. Functional diversity is a complex function of resource availability largely independent of primary production.
3. The system's metabolic activity exhibits an overall downward trend towards late succession and is higher than predicted by allometric relationships during a period of overexploitation of prey resources.
4. Eco-exergy is unsuitable to characterize successional progress in ecosystems, if species replacement does not lead to higher organismic complexity in terms of their proteome information.
5. The trophic transfer efficiency, the system metabolic activity, and the functional diversity form a minimal set of key indices which combine to a meaningful picture of successional progress, and are relatively well available from empirical data. Indices based on measurements of the flow of matter and energy strongly add to the mechanistic understanding of successional progress. In contrast to ascendancy, the weighted connectance is such an informative index because it is independent from the total system throughput and captures the effect of the increasing flow diversity across several trophic levels.

Our study helps to improve the mechanistic understanding of successional progress with the future vision to successfully manage ecological succession in systems undergoing environmental change [1], [175]. We hope that our study stimulates further analyses of empirical data sets and that ecologists may use our proposed set of system-level indices as a benchmark to quantify successional progress in other ecosystems.

## Supporting Information

Text S1 Supporting information text.
(DOC)

Data S1 Data collection from Lake Constance.
(XLSX)

Video S1 Video animation of plankton succession in Lake Constance.
(MPG)
