# Supplementary material for: Benchmarking Successional Progress in a Quantitative Food Web
Source: PLoS One. 2014 Feb 27;9(2):e90404. doi: 10.1371/journal.pone.0090404 (PMC3937422; doi:10.1371/journal.pone.0090404)
Supplement: Text S1 — Supporting information text. (DOC) [file pone.0090404.s001.doc]

# S1 Text: Supporting Information

# Boit, U. Gaedke: Benchmarking successional progress in a quantitative food web

*24-guilds resolution of the LC food web*

The phytoplankton were divided into 6 guilds (cf. Table 1 in main text). Guilds 1 to 5 are eukaryotes labelled **Alg1** through **Alg5**. Alg1 and Alg5 comprise small and well-edible phytoplankton species, while Alg2-4 consist of larger, less edible phytoplankton or species that form colonies. Guild 6 represents the autotrophic picoplankton including single cell cyanobacteria labelled **APP**. Guild 7 is labelled **Bac** and contains all bacterial strains other than autotrophic cyanobacteria. The zooplankton were divided into 13 guilds numbered 8 to 20. Guild 8 includes heterotrophic nanoflagellates labelled **HNF**. Guilds 9 to 13 are ciliates which are small protozoan grazers labelled **Cil1** to **Cil5**. Guilds 14 to 17 are medium-sized, metazoan grazers called rotifers. Guilds 14 to 16 vary in body mass and feeding strategies and are labelled **Rot1** to **Rot3**. Guild 17 comprises the larger rotifer genus *Asplanchna* *spp.* labelled **Asp**. Guild 18 labelled **Dap** includes predominantly herbivorous crustaceans (cladocerans such as daphnids, *Bosmina* and the calanoid copepod *Eudiaptomus*) which are omnivorous intraguild predators reproducing fast (e.g. by parthenogenesis) at high food availability. Dap share their phytoplankton prey species with faster metabolizing, small grazers (ciliates and rotifers) which adult Dap eat. Dap share almost all their heterotrophic resource species with guild 19 which includes predominantly carnivorous crustaceans (cyclopoid copepods) labelled **Cyc**. Guild 20 is labelled **Lep** and includes the large carnivorous *Leptodora* and *Bythotrephes*. The fishes were grouped into fish larvae and juveniles in guilds 21 and 22, respectively, planktivorous fish in guild 23, and piscivorous fish in guild 24.

**Table S1. Binary network indices of the 24-guilds LC food web.**

| **Index** | **Abbrev.** | **Values** |
| --- | --- | --- |
| Number of guilds | S | 24 |
| Links per guild | L/S | 4.54 |
| Connectance | L/S^2^ | 0.19 |
| Fraction top predators | - | 0.08 |
| Fraction intermediate guilds | - | 0.63 |
| Fraction basal guilds | - | 0.29 |
| Frac. herbivorous | - | 0.08 |
| Generality STDEV^1^ | GenSD | 1.08 |
| Vulnerability STDEV^1^ | VulSD | 0.59 |
| Connectivity STDEV^1^ | ConnSD | 0.54 |
| Mean Short-weighted trophic level^1^ | SWTL | 2.12 |
| Fraction omnivorous | - | 0.42 |
| Fraction cannibalistic | - | 0.04 |
| Characteristic Path Length | $\overline{D_{bin}}$ | 1.61 |
| Normalized char. path length | *D_norm_* | 0.77 |
| Mean cluster coefficient | $\overline{Q_{w}}$ | 0.56 |
| Normalized mean cluster coeff. | *Q_norm_* | 2.96 |

# Binary network indices hardly changed during succession because all guilds were almost always present in the network. They are given here for a general overview of structural food web properties and to enable a comparison with published binary webs. ^1^Indices as defined by Williams and Martinez [1].

# Table S2: Spearman rank correlation coefficients *r_s_* between time series of system-level indices in LC.

| **Index** | | ***H_bio_*** | ***SSS*** | ***C_w_*** | ***PP*** | ***P_tot_/B_tot_*** | ***TE*** | ***Avg. body mass*** |
| --- | --- | --- | --- | --- | --- | --- | --- | --- |
| *H_bio_* | | 1 | -0.07 | 0.5^***^ | 0.39^***^ | -0.17^**^ | -0.3^***^ | -0.56^***^ |
| *SSS* | |  | 1 | 0.6^***^ | -0.37^***^ | -0.51^***^ | 0.92^***^ | 0.71^***^ |
| *C_w_* | |  |  | 1 | -0.38^***^ | -0.81^***^ | 0.51^***^ | 0.37^***^ |
| *PP* | |  |  |  | 1 | 0.79^***^ | -0.55^***^ | -0.71^***^ |
| *P_tot_/B_tot_* | |  |  |  |  | 1 | -0.57^***^ | -0.53^***^ |
| *TE* | |  |  |  |  |  | 1 | 0.87^***^ |
| *Avg. body mass* |  | |  |  |  |  |  | 1 |

The indices are (cf. Table 3 in main text): *PP* = primary production, *TE* = trophic transfer efficiency, *P_tot_/B_tot_* = system’s mass-specific metabolic activity, *H_bio_* = functional diversity, *C_w_* = weighted connectance, *SSS* = Slope of the normalized biomass size spectrum, *Avg. body mass* = average body mass within the plankton community. ^***^significant at α = 0.001, ^**^significant at α = 0.01.

**
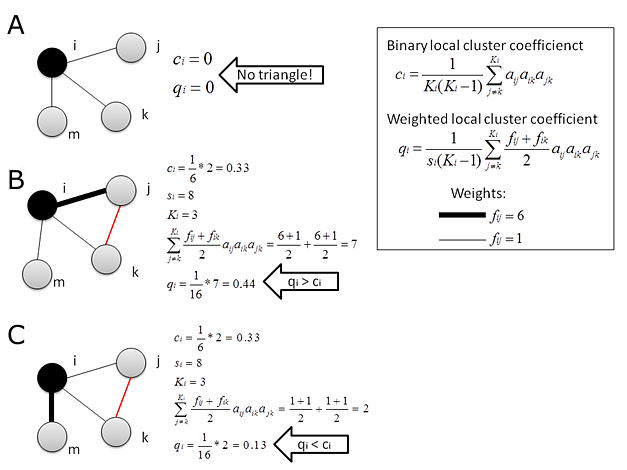
**

**Fig. S1: Example calculation of the weighted cluster coefficient.** Node *i* is the focal node for which the weighted local cluster coefficient *q_i_* is calculated [2]. The binary local cluster coefficient *c_i_* is shown for comparison and can be equal to (A), smaller than (B), or greater than (C) its weighted counterpart. Note that only the weights between the focal node *i* and its direct neighbors *j*, *k*, and *m* are relevant in the calculation of *q_i_*. The link between *j* and *k* (highlighted red) is only relevant for the adjacency coefficients (*a_ij_*, *a_ik_*, and *a_jk_* must all be 1 for the sum over *K* to be > 0) that indicate if a triangular constellation exists. The average across all local *q_i_* is the weighted average cluster coefficient of the network $\overline{Q_{w}}= 1/(S\sum_{i}^{S} q_{i})$ with *S* = number of nodes.

**
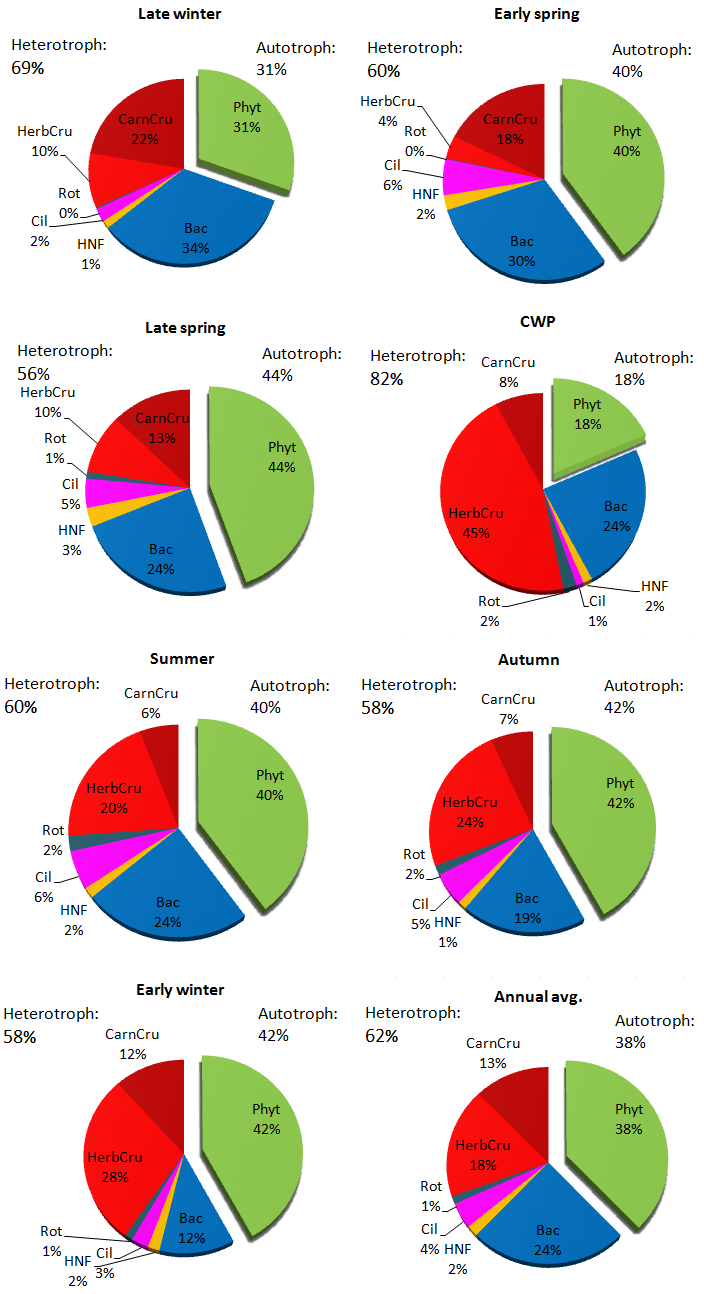
**

**Fig. S2: Biomass distributions in units of carbon within the plankton community in LC.** Based on the same data as Fig. 1A-B in the main text, the diagrams show the relative biomass contributions averaged across the respective phase and the annual average across phases 2-6. The annual avg. was weighted with the avg. number of days per phase to account for variability in phase length. Phase weights: Late winter: 21%; Early spring: 10%; Late spring: 12%; CWP: 6%; Summer: 24%; Autumn: 13%; Early Winter: 13%.


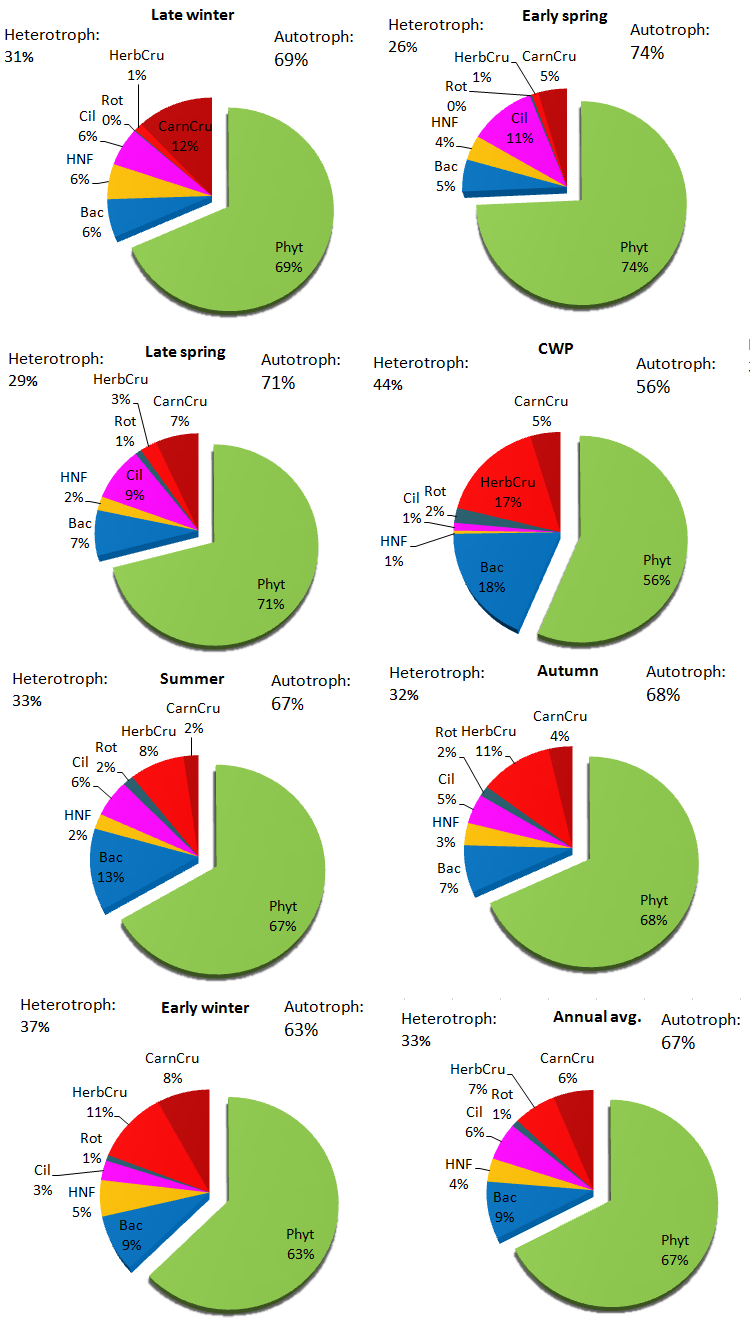


**Fig. S3: Production distributions in units of carbon within the plankton community in LC.** Based on the same data as Fig. 1C-D in the main text, the diagrams show the relative production contributions averaged across the respective phase and the annual average across phases 2-6. Phase weights for the annual average as in Fig. S2.


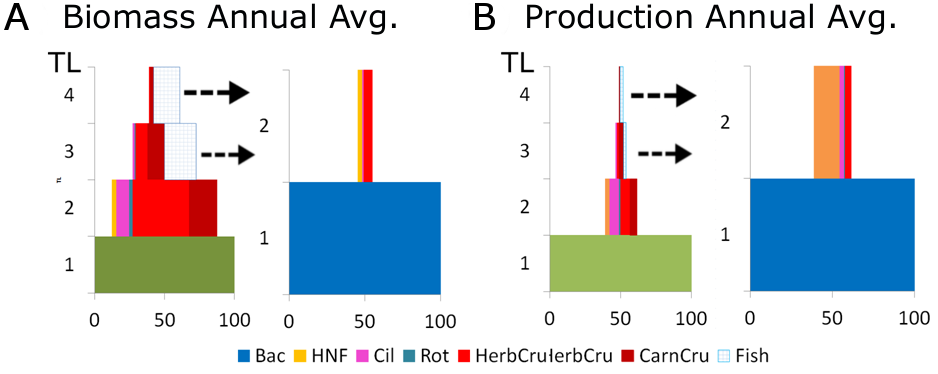


**Fig. S4: Annual averages of biomass (a) and production (b) pyramids.** Biomass and production were based on the same data as Fig. 1C-D in the main text. The biomass on trophic level 2 (trophic level 3) is approx. 75% (45%) of the biomass on trophic level 1. Given that fish biomass (production) on trophic level 3-4 was approximated due to commercial exploitation of fish in LC, the biomass (production) on trophic level 3-4 is underestimated in this illustration. Overall, the shape of the biomass pyramid is consistent with the slope of the normalized biomass size spectrum of approx. -1 (see Methods). Note that the production of bacterivores in the detritus chain (B) is considerably higher than their biomass (A). This implies high metabolic activity of bacterivores in comparison to the bacteria, while much of bacterivores’ biomass is consumed. Phase weights as in Fig. S2.


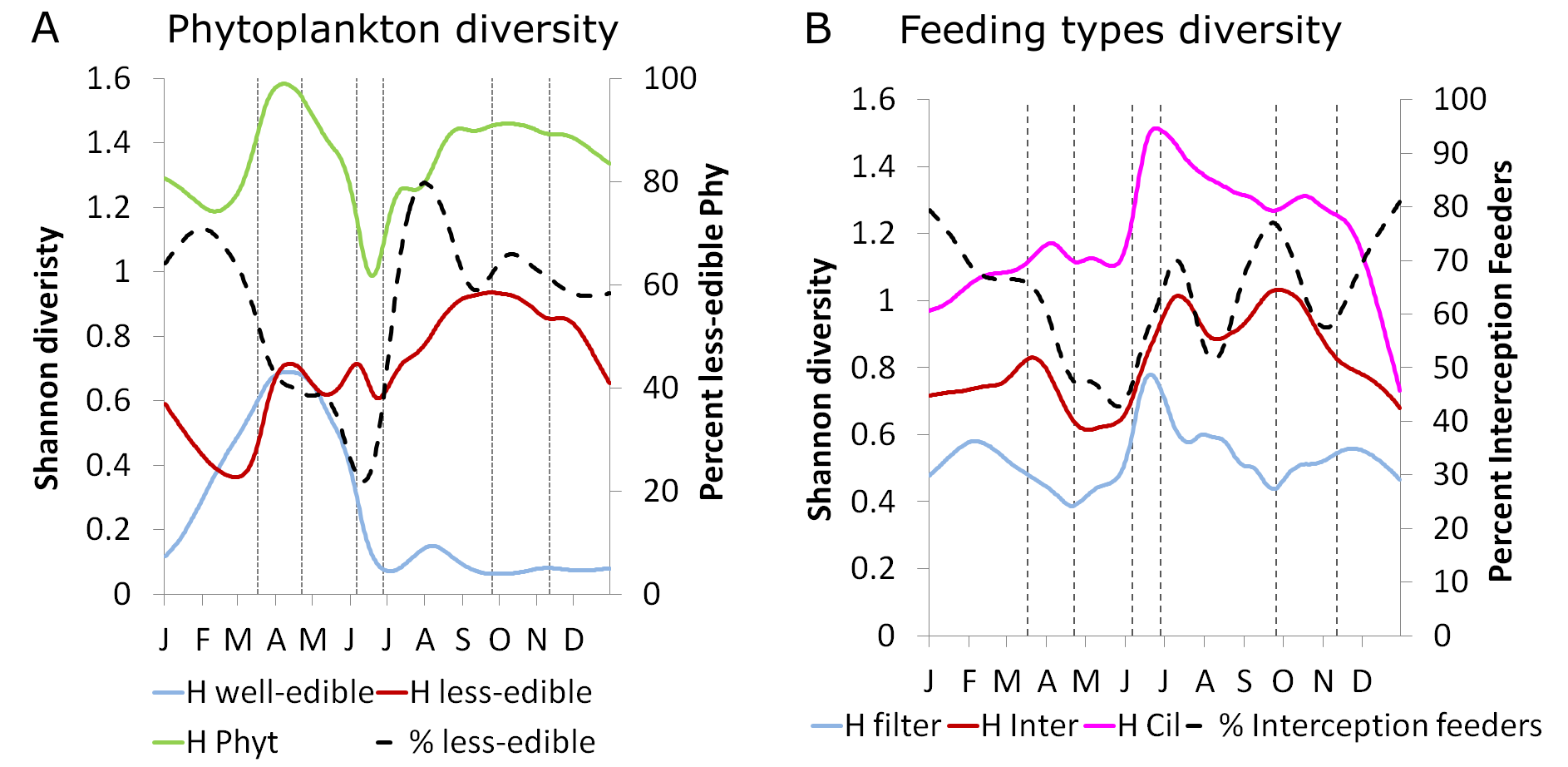


**Fig. S5. Functional diversity indices at the functional group level.** (A) Functional diversity (primary axis) of all phytoplankton guilds *H_Phyt_* (same as in Fig. 4A in the main text) was subdivided into *H_well-edible_* of the the well-edible and *H_less-edible_* of the less-well edible eukaryotic phytoplankton. The percentage of less-edible phytoplankton biomass (secondary axis) increased during succession. (B) Functional diversity (primary axis) of unicellular grazers *H_Cil_* (Cil = ciliates). The *H_filter_* of non-selective filter feeders among the ciliates was maximal during the CWP, whereas the *H_inter_* of more specialized interception feeders increased during succession. The relative biomass of interception feeders (secondary axis) also increased during succession.

**
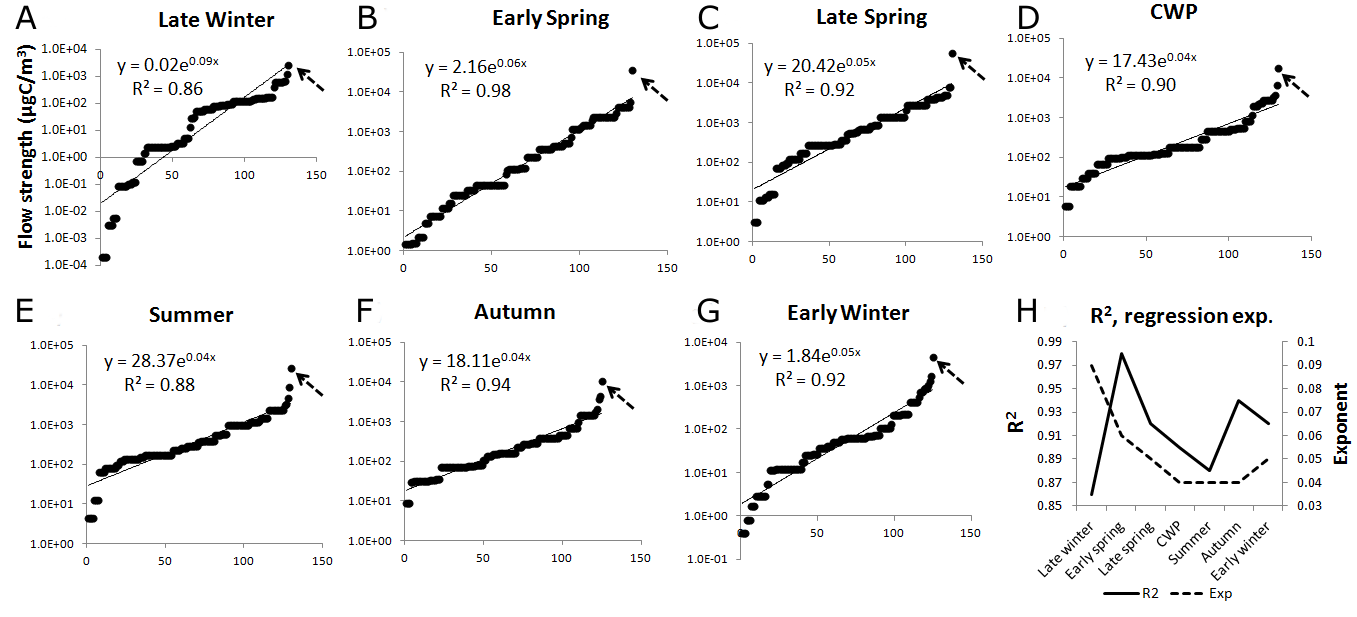
**

**Fig. S6: Carbon (C) flow distributions of feeding links during the seasonal succession.** Flows are based on the 24-guilds resolution derived from the 8-groups resolution (see Methods). Most feeding links (C-flows) were weak (note the logarithmic scale of the y-axis) and only a few dominated in winter (A, G), indicated by a steep slope (higher regression exponent) of the trendline (H). Bacteria consuming detritus was the largest trophic flow in all seasons (arrows). The flow strengths increased during the spring bloom (B-C). This was reflected in a higher intercept of the trendline. Strong flows developed between phytoplankton and herbivorous crustaceans during the CWP (D). The flow distribution became more even in summer and autumn (E-F), reflected in the smallest slope of the trendline and intermediate scatter (R^2^ = 0.94) around the trendline (and by the higher flow diversity, cf. Fig. S8 and Fig. 6E in the main text). R^2^ was maximal in early spring indicating the lowest scatter around the regression line because the weakest flows observed in winter had already strengthened, but the strong flows to the herbivorous crustaceans had not yet developed (H).


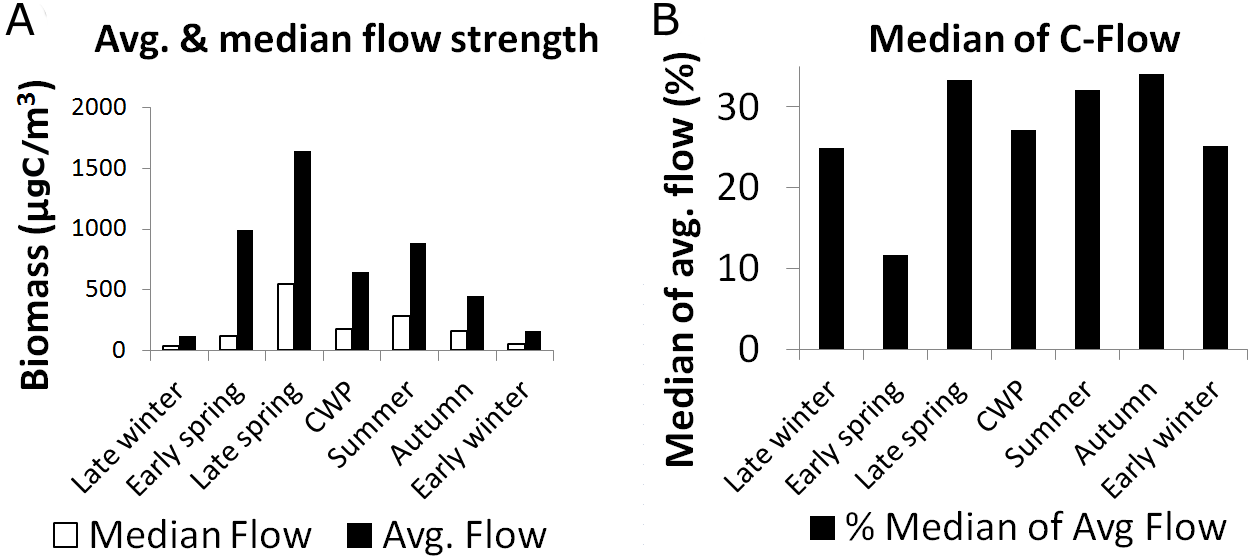


**Fig. S7: Carbon (C) flow distributions of feeding links in LC.** (A) Avg. C-flow strength compared to median flow strength during succession based on the 24-guilds resolution derived from the 8-groups resolution (see Methods). The median flow was always smaller than the avg. flow indicating a skewed flow strength distribution with a few strong flows and a long tail towards the small ones (cf. Fig. S6). (B) Same data as in (A), showing the ratio (%) between median and avg. flow. Prior to the phytoplankton bloom in early spring, the median was only about 10% of the average flow. During the spring bloom and throughout summer and autumn, the median increased to about 30% of the avg. flow.


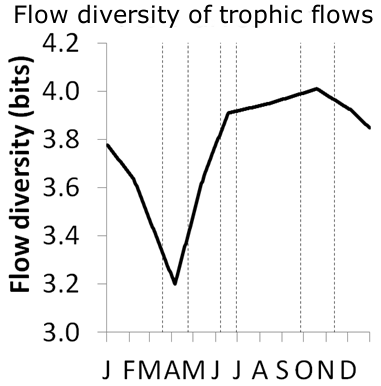


**Fig. S8: Flow diversity of the trophic C-flows in the 8-group resolution.** The temporal pattern of the flow diversity was closely correlated with weighted connectance *C_w_* (see Fig. 6A in the main text), if the set of links considered in the calculation only included the trophic flows within the 8-groups network and the detritus pool, but excluded external system in- and outputs, biomass storage flows, and respiration. The pattern of the flow diversity *H_flow_* as it was investigated in the main text (cf. Fig. 6E and see above for the equation) remained qualitatively similar when these additional flows were also included in the total system throughput (*TST*) according to Ulanowicz ([3]. The latter definition of *TST* was used for the calculation of the relative ascendency *Asc_rel_* (see Methods).


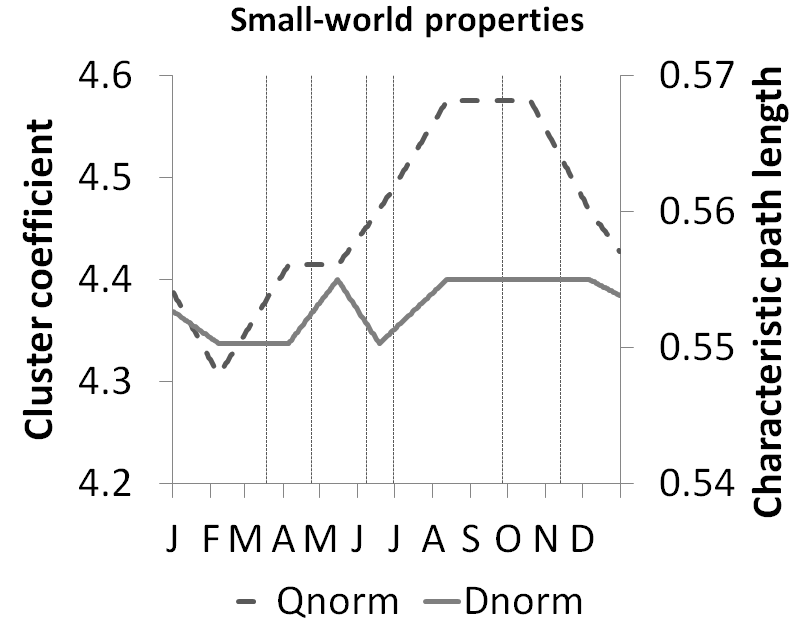


**Fig. S9: Weighted cluster coefficient and weighted characteristic path length based on the 8-groups resolution.** The cluster coefficient showed an increasing trend in the lower 8-groups resolution. The more detailed pattern observed in the 24-guilds resolution (cf. Fig. 6B in the main text) is lost due to the bundling of the energy links. The temporal pattern of the weighted characteristic path length remained almost constant through time.

**
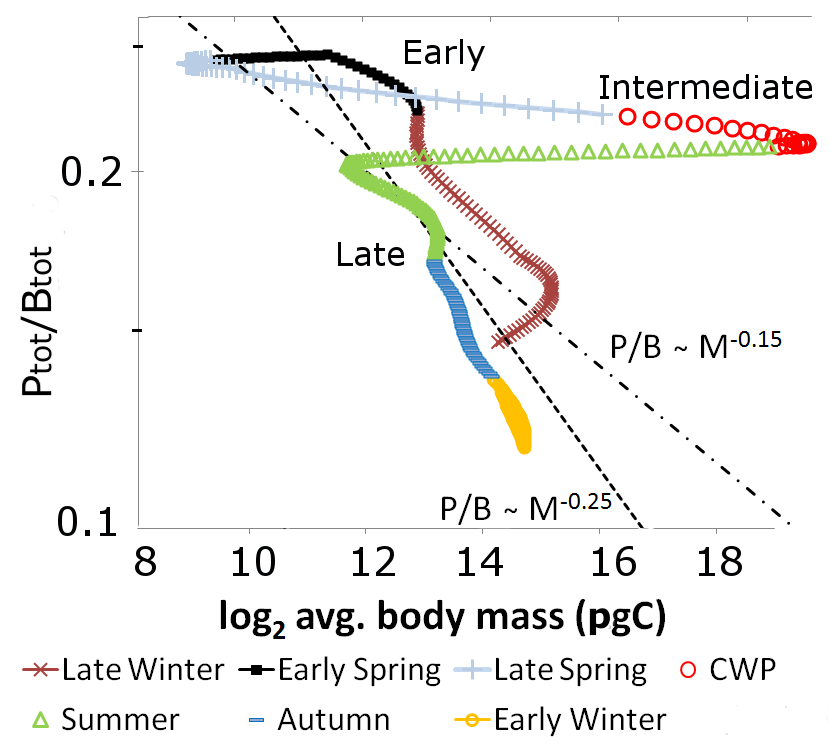
**

**Fig. S10: System metabolic activity versus average body mass.** During early and intermediate succession, the system’s mass-specific metabolic activity (P_tot_/B_tot_ = total production/total biomass) was higher than predicted by the allometric scaling rule *P/B* ∝ *M^-0.25^* (dotted line) or *P/B* ∝ *M^-0.15^* (dashed-dotted line) for plankton systems [4]. The intercepts of these two idealized lines were chosen for better comparability between the theoretically predicted and the observed slopes in LC.

References

1. Williams RJ, Martinez ND (2004) Limits to trophic levels and omnivory in complex food webs: theory and data. American Naturalist 163: 458-468.

2. Barrat A, Barthelemy M, Pastor-Satorras R, Vespignani A (2004) The architecture of complex weighted networks. Proceedings of the National Academy of Sciences of the United States of America 101: 3747-3752.

3. Ulanowicz RE, Norden JS (1990) Symmetrical Overhead in Flow Networks. International Journal of Systems Science 21: 429-437.

4. de Castro F, Gaedke U (2008) The metabolism of lake plankton does not support the metabolic theory of ecology. OIKOS 117: 1218-1226.
